# Neoadjuvant apatinib addition to sintilimab and carboplatin-taxane based chemotherapy in patients with early triple-negative breast cancer: the phase 2 NeoSAC trial

**DOI:** 10.1038/s41392-025-02137-7

**Published:** 2025-02-07

**Authors:** Guoshuang Shen, Zhilin Liu, Miaozhou Wang, Yi Zhao, Xinlan Liu, Yujin Hou, Wenbiao Ma, Jingqi Han, Xiaofeng Zhou, Dengfeng Ren, Fuxing Zhao, Zitao Li, Shifen Huang, Yongzhi Chen, Yingjian He, Yan Liu, Zijun Zhu, Yongxin Li, Jinming Li, Mengting Da, Hongnan Mo, Feng Du, Liang Cui, Jing Bai, Zhen Liu, Fei Ma, Jiuda Zhao

**Affiliations:** 1 Breast Disease Diagnosis and Treatment Center, Affiliated Hospital of Qinghai University, Affiliated Cancer Hospital of Qinghai University, Xining, China; 2 Medical Oncology Department of General Hospital of Ningxia Medical University, Yinchuan, China; 3 Department of Oncology, 2nd Ward, Qinghai Provincial People’s Hospital (Breast and Thyroid Surgery), Xining, China; 4 Department of pathology, Affiliated Hospital of Qinghai University, Affiliated Cancer Hospital of Qinghai University, Xining, China; 5 Key laboratory of Carcinogenesis and Translational Research (Ministry of Education/Beijing), Breast Center, Peking University Cancer Hospital & Institute, Beijing, China; 6 Department of Public Health, Medical College of Qinghai University, Xining, China; 7 State Key Laboratory of Molecular Oncology, Department of Medical Oncology,National Cancer Center/National Clinical Research Center for Cancer/Cancer Hospital, Chinese Academy of Medical Sciences and Peking Union Medical College, Beijing, China; 8 Key Laboratory of Carcinogenesis and Translational Research (Ministry of Education/Beijing),The VIPII Gastrointestinal Cancer Division of Medical Department, Peking University Cancer Hospital and Institute, 52 Fucheng Rd, Haidian District, Beijing, China; 9 Geneplus-Beijing Institute, Beijing, China

**Keywords:** Breast cancer, Cancer microenvironment, Drug development

## Abstract

We aimed to evaluate the efficacy and safety of adding apatinib, to sintilimab and chemotherapy in the neoadjuvant treatment of early triple-negative breast cancer (TNBC). In the phase 2 NeoSAC trial, patients with early TNBC received six cycles of apatinib, sintilimab, nab-paclitaxel, and carboplatin followed by surgery. The primary endpoint was pathological complete response (pCR) rate. Specimens collected pre-neoadjuvant therapy and post-surgery were retained for comprehensive analysis of predictive biomarkers and the impact on the tumor microenvironment. Among 34 enrolled patients, 24 achieved pCR (70.6%; 95% confidence interval (CI), 53.0-85.3), and 79.4% (95% CI, 65.1-93.7) had residual cancer burden 0-I. Imaging evaluation showed 21 complete responses (61.8%) and 13 partial responses (38.2%). The most common grade 3-4 adverse events were leukopenia (47%), neutropenia (36%), and thrombocytopenia (24%). The 36-month disease-free survival rate stood at 94.1% with a median follow-up of 39.1 months. Notably, baseline high ImmuneScore, immune cell infiltration, and enrichment of interferon-related pathways correlated with pCR. Comparison of pre-neoadjuvant and post-surgery data revealed that the pCR group treated with this novel regimen exhibited an upregulation of distinct immune cell subsets, thereby activating the tumor microenvironment. Moreover, higher oxeiptosis scores were associated with an increased likelihood of achieving pCR. Following neoadjuvant therapy, the pCR group showed a decrease in oxeiptosis score, whereas the non-pCR group exhibited an increase. Our study suggests that apatinib, sintilimab combined with carboplatin and nab-paclitaxel chemotherapy showed a promising clinical activity and manageable safety profile in early TNBC and merits further study. ClinicalTrials.gov registration: NCT04722718.

## Introduction

Triple-negative breast cancers (TNBC) represent a highly heterogeneous and aggressive subtype, associated with a higher risk of recurrence, distant metastases, and poorer prognosis compared to other subtypes.^1,2^ However, in the neoadjuvant setting, early TNBC usually exhibits high response rates to chemotherapy, leading to a significantly improved pathological complete response (pCR) rate and long-term survival outcomes.^1,3^ Neoadjuvant treatment plays an increasingly important role in early TNBC to down staging and improve survival.^1,3^ Traditionally, anthracyclines and taxanes are preferred neoadjuvant treatments for TNBC. However, recent research has introduced promising alternatives, including platinum-based chemotherapy, immune checkpoint inhibitors (ICIs), Poly (ADP-ribose) polymerase inhibitors, antiangiogenic therapies, and antibody-drug conjugates, broadening the range of treatment options available for TNBC.^1,2^

Among new neoadjuvant treatments, platinum-based agents significantly increase pCR rates, recommended to varying degrees in international guidelines.^1,2^ ICIs, especially pembrolizumab, show excellent efficacy, dramatically improving pCR rates and long-term survival,^4,5^ also endorsed in recent guidelines. Adding ICIs to neoadjuvant treatments notably boosts pCR rates in TNBC, but their efficiency needs enhancement. In KEYNOTE-522 and IMpassion031 trials, when ICIs combine with chemotherapy, pCR rates in programmed death-ligand 1 (PD-L1)–negative population are only 45.3% and 48%, respectively, indicating majority unable to achieve pCR. Even in PD-L1–positive population, the pCR rate in a minority of patients can also be further increased as their pCR rates are 68.9% and 69%, respectively.^5,6^ Moreover, neoTRIP trial failed to show pCR benefit with addition of ICIs to carboplatin-taxane-based chemotherapy, with pCR rates of 48.6% and 44.4% in ICIs arm and placebo arm, respectively.^7^ These results suggest a subset of TNBC resistant to chemotherapy with or without ICIs in neoadjuvant setting, needing to be overcome for further improvement in pathological response and survival.

Tumor angiogenesis and immunomodulation interact closely. The abnormal tumor vasculature polarizes inflammatory cells toward immune suppression through hypoxia and acidosis of the tumor microenvironment and pro-angiogenic cytokine alter the proliferation, differentiation, and function of immune cells. While suppressive immune cells can also induce angiogenesis. Such intersection between tumor angiogenesis and immune suppression result in a vicious cycle that disturbs the outcomes of antitumor immunotherapy.^8,9^ Therefore, enhancing antitumor immunotherapy by normalizing tumor vessels with antiangiogenic agents holds significant promise.^8^ This approach has shown benefits not only in preclinical studies using breast cancer mouse models but also in clinical trials involving metastatic TNBC.^10,11^ For instance, antiangiogenic therapy can enhance the effectiveness of ICIs by upregulating PD-L1 expression and promoting the infiltration and activation of CD8 + T cells within the tumor microenvironment in breast cancer mouse models.^10^ Moreover, the combination of antiangiogenic therapy and ICIs has demonstrated synergistic antitumor effects, achieving an objective response rate of 81.3% as a first-line treatment for advanced TNBC.^11^

Herein, we conducted the phase 2 NeoSAC trial to explore the efficacy, safety, and biomarker of the addition of apatinib, a vascular endothelial growth factor receptor 2 (VEGFR2) tyrosine kinase inhibitor, to sintilimab, an anti–programmed death 1 antibody, plus carboplatin-taxane based chemotherapy in patients with early TNBC.

## Results

### Patients

From Jan 26, 2021 to Aug 19, 2023, 52 patients were screened, with 34 enrolled across three institutions (Supplementary Fig. 1). Among the 16 patients in the first stage of the trial, 12 achieved pCR, prompting enrollment of 18 additional patients in the second stage. The overall landscape of the study results is presented in Supplementary Fig. 2.

The median age was 49.5 years (range 27-70), with all cancers exhibiting invasive ductal histology. Of the enrolled patients, 23 (67%) had clinical T2 tumors, while 11 (33%) had T3-4a/b tumors; 65% presented with axillary node involvement. Overall, 22 patients were classified as stage II (64%) and 12 as stage III (36%) (Table 1). Mastectomy was performed in 26 patients (76%), while seven (21%) underwent breast conserving surgery.

**Table 1:** Patient characteristics

Characteristic	All patients
Median age (range), years	49.5 (27–70)
Sex	
Female	34 (100%)
Menopausal status	
Premenopausal	17 (50%)
Postmenopausal	17 (50%)
Histology	
Invasive ductal carcinoma	34 (100%)
Other	0
ECOG performance status	
0	32 (94%)
1	2 (6%)
Clinical tumor size	
cT1	0
cT2	23 (67%)
cT3	6 (18%)
cT4a-b	5 (15%)
Clinical node stage	
cN0	12 (35%)
cN1	18 (53%)
cN2	4 (12%)
Clinical stage at diagnosis*	
IIA	10 (29%)
IIB	12 (35%)
IIIA	7 (21%)
IIIB	5 (15%)

Data are presented as median (range), or n (%). *ECOG* Eastern Cooperative OncologyGroup. *Classifed on the basis of on the American Joint Committee on Cancer 8th edition staging system

### Efficacy

**Fig. 1:**
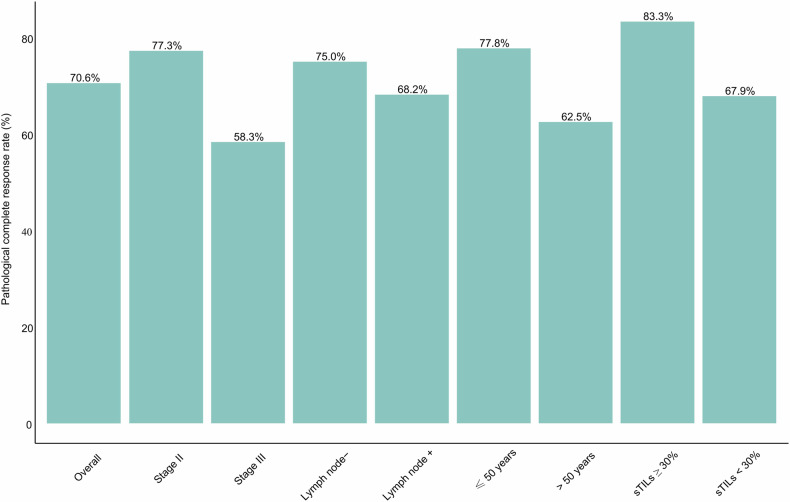
Pathological complete response rates overall and in subgroups. sTILs stromal tumor-infiltrating lymphocytes

**Fig. 2:**
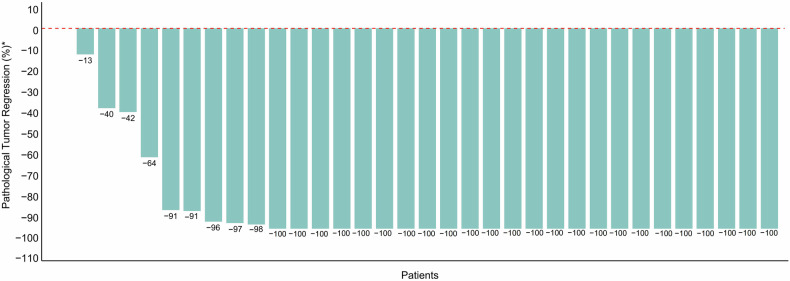
Pathological tumor regression in the per-protocol population. *The percentage of pathological tumor regression per tumor among the 33 per-protocol patients that could be evaluated for a pathological response

**Fig. 3:**
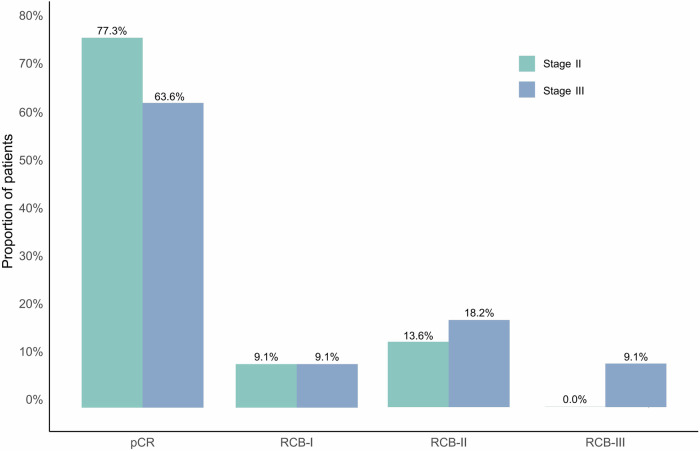
Residual cancer burden according to different stage in the per-protocol population. RCB residual cancer burden; pCR pathological complete response

In the overall 34 intention-to-treat population, 33 participants who were evaluable for response could be assessed for pCR in both breast and nodes except one patient lost to follow up, 24 (70.6%; 95% CI, 53.0-85.3) of whom achieved pCR (ypT0/Tis, N0) (Fig.1, Supplementary Table 1). Similar results were observed in the 33 per-protocol population with a pCR rate of 72.7% (95% CI, 57.6-87.9) (Fig. 2, Supplementary Table 1). The breast pCR was 73.6% (95% CI, 55.9-88.2) and 75.8% (95% CI, 60.6-87.9) in intention-to-treat population and per-protocol population, respectively. The rate of residual cancer burden (RCB) 0-I was 79.4% (95%CI, 65.1-93.7) in the intention-to-treat population and 81.8% (95%CI, 67.9-95.7) in the per-protocol population (Supplementary Table 1). Based on the RCB analysis, pCR was achieved in 77.3% of stage II patients, surpassing the 63.6% in stage III. Both stages had 9.1% in RCB-I. Stage III patients had higher proportions in RCB-II (18.2% vs. 13.6%) and were the only group with RCB-III (9.1%) (Fig. 3).

Subgroup analyses showed higher pCR rates in patients with stage II compared to stage III (77.3% vs 58.3%), age ≤ 50 years versus > 50 years (77.8% vs 62.5%), nodal-negative versus nodal-positive (75.0% vs 68.2%), and stromal tumor-infiltrating lymphocytes (sTILs) ≥ 30% versus sTILs < 30% (83.3% vs 67.9%) (Fig. 1). These results suggest a relationship between certain clinical and pathological features and pCR outcomes. Specifically, patients with earlier-stage disease (stage II), younger age, nodal-negative status, and higher levels of sTILs tended to have higher pCR rates.

All patients in the intention-to-treat group underwent bilateral breast MRI at baseline and during neoadjuvant therapy assessment. 21 (61.8%) achieved complete response, while 13 (38.2%) had partial response, yielding an overall objective response rate (ORR) of 100.0% (Supplementary Table 1).

All 34 evaluated patients experienced at least one treatment-related adverse event. (Table 2). Prior to the carboplatin dose reduction amendment, 9 patients received AUC 2.0 of carboplatin, while 25 received AUC 1.5 thereafter. The most common grade 3–4 adverse events included leukopenia (16 [47%]), neutropenia (12 [36%]), and thrombocytopenia (8 [24%]). Possible immune-related adverse events, such as thyroid dysfunction (9 [26%]), rash (6 [18%]), and fever (3 [9%]), were all grade 1 or grade 2. No treatment-related deaths or pneumonitis occurred.

**Table 2:** Treatment-related adverse events during neoadjuvant treatment in the safety population (n = 34)

	Patients, No. (%)		
Adverse events	Any treatment-related adverse event	Grade 3 adverse events	Grade 4 adverse events
Total		25 (74%)	6 (12%)
Hematologic			
Thrombocytopenia	29 (85%)	6 (18%)	2 (6%)
Leukopenia	29 (85%)	14 (41%)	2 (6%)
Anemia	23 (68%)	2 (6%)	0
Neutropenia	22 (65%)	8 (24%)	4 (12%)
Febrile neutropenia	11 (32%)	5 (15%)	0
Nonhematologic			0
Alopecia	34 (100%)	0	0
Fatigue	25 (74%)	0	0
Hypoalbuminemia	16 (47%)	0	0
Peripheral sensory neuropathy	18 (53%)	0	0
Proteinuria	12 (35%)	0	0
Anorexia	11 (32%)	0	0
Hypertension	10 (29%)	2 (6%)	0
Pain	9 (26%)	1 (3%)	0
Hyperglycemia	8 (24%)	1 (3%)	0
ALT elevation	7 (21%)	3 (9%)	0
AST elevation	7 (21%)	4 (12%)	0
Hand-foot syndrome	7 (21%)	0	0
Nausea	23 (68%)	0	0
Arthralgia	5 (15%)	0	0
Edema	5 (15%)	0	0
Mucositis oral	4 (12%)	0	0
Vomiting	8 (24%)	0	0
Headache	2 (6%)	0	0
Syncope	2 (6%)	0	0
Immune related			
Thyroid dysfunction	9 (26%)	0	0
Rash	6 (18%)	0	0
Fever	3 (9%)	0	0

Data are presented as n (%)

*ALT* Alanine aminotransferase, *AST* Aspartate aminotransferase

No grade 5 treatment-related adverse events occurred

### Adverse events

Overall, 30 (88%) patients completed the scheduled 6 cycles of neoadjuvant therapy, with 30 (88%) receiving 6 cycles of apatinib and 28 (82%) receiving 6 cycles of sintilimab. One patient was lost to follow-up after three cycles, while one completed 4 cycles and two completed 5 cycles due to grade 3 adverse events and COVID-19. Fifteen (44%) patients experienced chemotherapy delays due to adverse events (7 [20%]) and COVID-19 (8 [24%]). Four (12%) patients required carboplatin dose modifications for grade 3 myelosuppression.

### Survival

As of November 25, 2024, with a median follow-up of 39.1 months (range: 28.1 to 47.5 months), one patient who achieved pCR passed away due to non-breast cancer-related causes and non-treatment-related adverse events. Another patient developed pulmonary metastasis, while the remaining patients continued to be free of recurrence or metastasis. The 36-month disease-free survival (DFS) rate was 94.1%.

### Biomarker

**Fig. 4:**
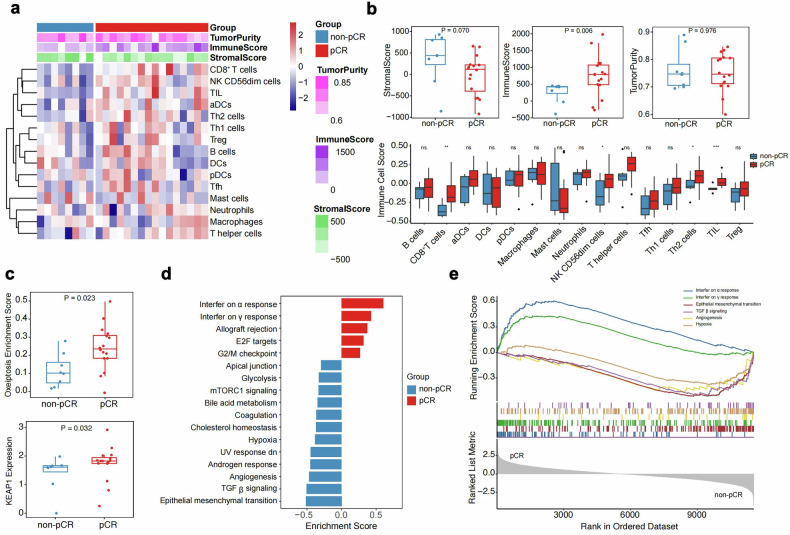
Baseline immune microenvironment comparison between the pCR and non-pCR groups. (**a**) Baseline samples between two groups of immune infiltrating cells and estimated stroma, immune cell, and tumor purity scores heatmap. (**b**) Comparison of differences in stromal, immune cell, tumor purity scores, and immune cell infiltration between groups. (**c**) Comparison of oxeiptosis scores and oxeiptosis-related genes. (**d**, **e**) Comparison of significantly different pathways. pCR, pathological complete response. ns, not significant. *, *p* < 0.05. **, *p* < 0.01. ***, *p* < 0.001

**Fig. 5:**
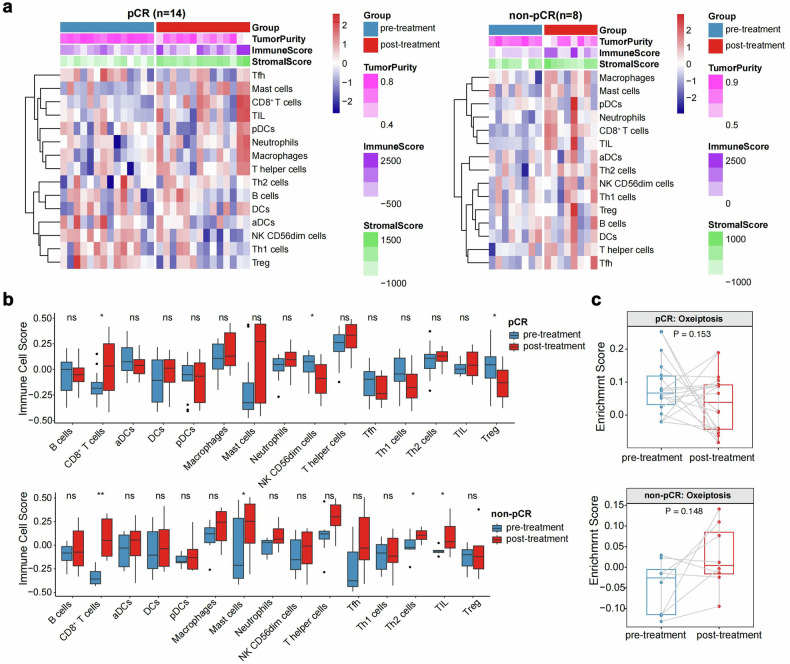
Comparison of dynamic changes in the immune microenvironment pre- and post-treatment between the pCR and non-pCR groups. (**a**) Comparison of differences in dynamic changes in immune infiltrating cells and estimated stroma, immune cell, and tumor purity scores pre-treatment and post-treatment heatmap. (**b**) Comparison of differences in dynamic changes in stroma, immune cell, tumor purity scores, and immune cell infiltration between pre- and post-treatment. (**c**) Comparison of dynamic changes in differences in oxeiptosis scores between pre- and post-treatment. pCR pathological complete response; ns, not significant. *, *p* < 0.05. **, *p* < 0.01

Among 33 patients undergoing surgery, we collected 24 pre-neoadjuvant therapy and 23 postoperative specimens. Reasons for specimen unavailability included 8 cases of COVID-19, 1 patient preference, and 1 postoperative specimen failing quality control. The RNA sequencing results showed that in 16 pCR and 8 non-pCR pre-neoadjuvant treatment specimens, higher ImmuneScore, interferon-related pathway enrichment, and oxeiptosis scores correlated with pCR (Fig. 4). In 14 pCR and 8 non-pCR matched pre- and post-neoadjuvant specimens, most immune cells increased after therapy in both groups. Treg cells decreased in the pCR group but increased in the non-pCR group. Additionally, although not statistically significant, the pCR group exhibited decreased oxeiptosis scores, while the non-pCR group showed increased scores post-therapy (Fig. 5). The multiplex immunofluorescence results further confirmed predictive efficacy and dynamic changes, including CD8, Treg, and CD56 cell density. Additionally, PD-L1-positive patients had a higher pCR rate, though not statistically significant. The density of CD31-negative CD8-positive cells in the tumor area correlated with pCR, and post-treatment, CD31-positive PD-L1-negative cell density decreased in the stroma of the pCR group but not in the non-pCR group (Supplementary Fig. 3, Supplementary Fig. 4).

## Discussion

To our knowledge, NeoSAC stands as the first clinical trial registered on ClinicalTrials.gov to explore the efficacy and safety of antiangiogenic therapy combined with immunotherapy alongside neoadjuvant chemotherapy in early TNBC. This regimen demonstrated promising and high activity, boasting a pCR rate of 70.6%, an RCB 0-I rate of 79.4%, an ORR of 100%, and a manageable toxicity profile. Considering pCR and less residual disease (RCB-1) after neoadjuvant therapy associated with excellent prognosis in TNBC,^3,4,12^ these results are particularly encouraging.

The regimen in NeoSAC mainly considers the efficacy of enhancing ICIs combined with chemotherapy through antiangiogenic therapy, as well as adopting appropriate chemotherapy protocols. Antiangiogenic therapy, which reverses resistance to ICIs and enhances their efficacy, has been validated in both preclinical and clinical studies of advanced breast cancer. When combining apatinib with ICIs, continuous dosing (250 mg orally from day 1 to day 14) of apatinib demonstrates a manageable safety profile and superior therapeutic effects compared to intermittent dosing (250 mg orally from day 1 to day 7) in advanced TNBC.^10^ Hence, we adopt apatinib with a dose of 250 mg oral from d1 to day 14 in this study. Sintilimab combined with antiangiogenic therapy also demonstrated synergistic activities in metastatic cervical cancer.^13^ In our study, the addition apatinib to sintilimab plus chemotherapy also conveyed that antiangiogenic therapy could increase the efficacy of immunotherapy and exhibited encouraging efficacy in early TNBC.

The chemotherapy regimen in NeoSAC is primarily designed based on efficacy, side effects, immune response activation, and appropriate treatment cycles. First, anthracycline and taxane-based neoadjuvant chemotherapy remain the standard of care for early TNBC. However, only approximately 30%–40% of patients achieve pCR, while platinum-based neoadjuvant chemotherapy significantly increases the pCR rate in TNBC.^14^ Concerns arise regarding the long-term survival benefit and hematological toxicities of platinum-based neoadjuvant chemotherapy. Considering most of included studies in that meta^14^ are sequential anthracycline and platinum agents even be used concurrently and likely leads to the bias of outcomes, and neoadjuvant chemotherapy is at least equivalent to long-term survival benefit compared with adjuvant chemotherapy, we performed a meta-analysis including randomized controlled trials that report survival of platinum-based chemotherapy compared with anthracycline and paclitaxel based chemotherapy in neoadjuvant or adjuvant setting in early TNBC. The results showed that platinum-based chemotherapy significantly improves both DFS and overall survival, regardless of whether platinum is combined with anthracycline. Additionally, most of side effects including hematological toxicities, liver enzyme and cardiac toxicity were more common in platinum combined with anthracycline except thrombocytopenia was more high in platinum without anthracycline chemotherapy.^15^ Such results are also reinforced by the BrighTNess study.^16^

Second, TONIC study demonstrated that both doxorubicin and cisplatin have capacity of inducing more favorable tumor microenvironment in TNBC.^17^ And nab-paclitaxel also not only improve both pCR rate and survival but also avoid the potential impact from immunosuppressive medications such as corticosteroid in early TNBC.^18^ Third, NeoCART study showed that 6 cycles of docetaxel plus carboplatin obviously increase pCR rate compared with 8 cycles of taxane- and anthracycline-based treatment (61.4% *vs* 38.6%).^19^ Hence, we adopt 6 cycles of chemotherapy with carboplatin plus nab-paclitaxel.

Studies investigating the addition of immunotherapy to neoadjuvant chemotherapy for early TNBC, such as KEYNOTE-522, GeparNuevo, Impassion031, and I-SPY2, all utilized 8 cycles of anthracycline-taxane-based chemotherapy, with pCR rates ranging from 53.4% to 64.8%.^4,6,20^ Notably, compared to these studies, our research shows an increase in the pCR rate. NeoTRIPaPDL1, the largest randomized controlled study using carboplatin-taxane-based, anthracycline-free neoadjuvant chemotherapy with longer cycles as 8, reported a relatively low pCR rate of 43.5% in 2019.^7^ Other studies, including NeoPACT,^21^ NCI-10013^22^ and cTRIO^23^ studies also report neoadjuvant ICIs combined with carboplatin and taxane resulted in a pCR rate of 57%, 57.8% and 56.5%, respectively. The pCR rate is significantly higher in this study than those three studies, though the patient’ clinical stage in NeoSAC was later than those three studies (stage II 64%, stage III 36% in NeoSAC *vs* stage II/III 88% in NeoPACT *vs* stage II 68.9%, stage III 31.1% in NCI-10013 *vs* stage II 67.7%, stage III 32.3% in cTRIO). The pCR rate in the NeoSAC study is also higher than the recently reported 64.5% in another study of neoadjuvant therapy combining anti-angiogenic, immunotherapy, and chemotherapy for TNBC, but it has a similar RCB 0-I rate of 80.6%.^24^ Although patients in that study underwent 8 cycles of chemotherapy, carboplatin was only administered for 4 cycles. Additionally, the use of the antiangiogenic drug anlotinib differed from the apatinib used in our study, potentially contributing to the differences in results. This result also strongly suggest that antiangiogenic therapy can significantly enhance the efficacy of ICIs combined carboplatin-taxane based chemotherapy. In addition, the extremely high rate of pCR and RCB 0-I of this regimen implies favorite prognosis.^3,12^ However, we acknowledge the need to address the discrepancies between our findings and the pCR rates reported in studies such as KEYNOTE-522 and NeoPACT. These differences may be attributed to several factors, including variations in chemotherapy regimens, the types of ICIs used, and differences in patient populations. To better understand the underlying reasons for these discrepancies, further studies are needed to compare different chemotherapy regimens, explore the impact of specific ICIs, and consider variations in patient characteristics.

There is also a potential concern about the roles of antiangiogenic therapy improving long-term outcome in this study. Whether bevacizumab have long-term outcome benefit for early TNBC remains unclear as previous studies demonstrate inconsistent outcome although it significantly increase pCR rates.^25^ Nevertheless, the neoadjuvant treatments among those studies are all based on bevacizumab combined with chemotherapy. It is obviously different with the regimen in this study that based on apatinib combined with ICIs and chemotherapy and the antiangiogenic therapy might have synergistic antitumor effects with both ICIs and chemotherapy. Moreover, antiangiogenic therapy can exactly sensitize ICIs treatment which can improve long-term survival when combined with chemotherapy in early TNBC. Additionally, a meta-analysis including 7 randomized controlled trials with 7491 patients also shows that bevacizumab mildly improves DFS (HR = 0.88, 95% CI, 0.78–0.98) but not overall survival (HR = 0.88, 95% CI, 0.77–1.01) who treated bevacizumab and standard chemotherapy as neoadjuvant or adjuvant treatment in early TNBC.^25^ At a median follow-up of 39.1 months, the 36-month DFS rate reached 94.1%, indicating the potential for improved survival with the NeoSAC regimen.

The safety profile of our study aligned with that of apatinib, sintilimab, carboplatin, nab-paclitaxel, or their combinations used for metastatic TNBC, as well as with ICIs plus neoadjuvant chemotherapy for early TNBC.^5,7,10,11,18,21,23^ The incidence of immune-related adverse events was generally lower compared to similar studies, which may be related to the drugs used. In the RCT,^26^ sintilimab was associated with a lower incidence of immune-related AEs. Additionally, the shorter chemotherapy regimen (only 6 cycles), the lower dose of carboplatin, and the smaller dose of apatinib in the treatment protocol could also contribute to this finding.

We conducted a comprehensive analysis of predictive biomarkers for pCR and the impact of neoadjuvant therapy on the tumor microenvironment. Overall, the RNAseq and multiplex immunofluorescence results are consistent. Elevated parameters of immune response, such as high ImmuneScore, immune cell infiltration, and enrichment of interferon-related pathways, correlated with pCR, while pathways like transforming growth factor beta (TGF-β) signaling and angiogenesis correlated with non-pCR. These findings are partially consistent with previous reports^27,28^ and suggest the potential utility of these biomarkers for predicting the efficacy of the NeoSAC regimen.

By comparing pre-neoadjuvant and post-surgery data for the aforementioned parameters, the pCR group treated with antiangiogenic therapy combined with ICI and chemotherapy exhibited a significant upregulation of distinct immune cell subsets, fully activating the tumor microenvironment. Similar but less pronounced phenomena were observed in the non-pCR group, indicating that this regimen can activate the tumor microenvironment, albeit with a more pronounced effect in the pCR group, while the non-pCR group still falls short of achieving optimal efficacy.

A significant finding of our study is that patients with higher oxeiptosis scores are more likely to achieve pCR. After neoadjuvant therapy, the pCR group exhibits a decrease in oxeiptosis score, while the non-pCR group shows an increase. Oxeiptosis is a novel form of reactive oxygen species-induced caspase-independent apoptotic cell death. Currently, there appear to be no direct reports on the relationship between oxeiptosis and antiangiogenic and ICI therapy. Nevertheless, research revealed that lloimperatorin triggers oxeiptosis in breast cancer cells, consequently restraining their proliferation and invasion.^29^ KEAP1 (Kelch-like ECH-associated protein 1) is known to regulate the Nrf2 pathway, which plays a critical role in oxidative stress responses and cellular survival mechanisms. While the relationship between KEAP1 and oxeiptosis remains largely unexplored, its involvement in modulating oxidative stress provides a compelling avenue for future investigation.^30^ Building on our current findings, our future research will focus on further exploring oxeiptosis by investigating its mechanistic pathways and assessing its role in therapeutic applications.

The present study is limited by the nonrandomized, small number of patients, and the lack of long-term follow up data, which influence drawing definitive conclusions. Sintilimab was also not administered in the adjuvant setting. Additionally, due to COVID-19, some samples couldn’t be collected, impacting our findings, particularly in the deconvolution of tumor microenvironment from bulk transcriptome data. Furthermore, a subset of patients was not tested for PD-L1, although it doesn’t predict ICIs efficacy in early TNBC. The biomarker analysis did not directly address key pathways such as EGFR or VEGF, which may be relevant to treatment outcomes. Future research should explore these pathways to provide a more comprehensive understanding of the molecular mechanisms.

In conclusion, the addition of apatinib to sintilimab and carboplatin-taxane-based neoadjuvant chemotherapy demonstrated promising clinical activity and well-tolerated safety in early TNBC. This study also identified efficacy-related biomarkers and demonstrated that this regimen can activate the immune microenvironment. Further investigation is warranted. We are planning a phase III trial to evaluate the addition of apatinib to immune checkpoint inhibitors and chemotherapy for neoadjuvant treatment in TNBC.

## Methods

### Study design and participants

The NeoSAC trial was an investigator-initiated, multicenter, single-arm, open-label, phase 2 study conducted at three hospitals in China (NCT04722718). It aimed to evaluate neoadjuvant apatinib in combination with sintilimab, carboplatin, and nab-paclitaxel chemotherapy in early TNBC. Eligible patients were aged 18–70 years with a histologically confirmed diagnosis of TNBC, defined as expression of estrogen (ER)/progesterone (PR) receptor-negative by immunohistochemistry (IHC) (<1% of tumor cells positive for ER/PR irrespective of staining intensity) and human epidermal growth factor receptor 2 (HER-2)-negative by IHC (0 or 1 + ) or fluorescence in situ hybridization. They had previously untreated, early-stage disease (clinical stage II and III, T stage: T1c, N stage: N1-2, or T stage: T2-4, N stage: N0-2), Eastern Cooperative Oncology Group performance (ECOG) status 0 to 1, and adequate organ function.

Key exclusion criteria were metastatic disease, bilateral or inflammatory breast cancer, and underwent tumor and/or lymph node excision biopsy prior to neoadjuvant therapy initiation. The trial protocol is available in the Supplementary Information.

This study obtained approval from the Ethics Committee of Affiliated Hospital of Qinghai University and each participating site (No. P-SL-2020078) and adhered to the guidelines of the Declaration of Helsinki and Good Clinical Practice. All patients provided written informed consent before enrollment.

### Procedures

Patients received apatinib 250 mg orally once daily from day 1 to day 14, sintilimab 200 mg intravenously on day 1, nab-paclitaxel 125 mg/m^2^ intravenously on day 1 and day 8, and carboplatin (AUC 1.5) on day 1 and day 8 of each 21-day cycle, for six cycles with mecapegfilgrastim support, prior to surgery. Due to frequent grade 3 neutropenia, carboplatin dose was reduced from AUC 2 to AUC 1.5 since May 2021.

Dose modification for apatinib and sintilimab was not allowed. Dose interruptions of these medications and modifications or interruptions of chemotherapy were permitted for any grade 3 or higher adverse events until the toxicity lessened to grade 1 or lower. Treatment could be interrupted for up to 2 weeks continuously within 3 weeks. Long-term outcomes were monitored after initiation of study therapy.

Clinical response was assessed every two cycles prior to surgery according to the Response Evaluation Criteria in Solid Tumors (RECIST) version 1.1. After surgery, local pathologists evaluated pathological complete response using the Miller-Payne scoring system and residual disease in nodes, along with RCB. Adverse events were monitored throughout the study and graded according to the National Cancer Institute Common Terminology Criteria for Adverse Events version 5.0.

### Outcomes

The primary endpoint was the pCR rate, defined as the absence of invasive residual disease in breast or nodes, with non-invasive breast residuals allowed (ypT0/Tis ypN0) post-neoadjuvant therapy. Secondary endpoints included breast pCR rate (ypT0/Tis), RCB rate, ORR, rate of breast-conserving surgery, and DFS, defined as the duration from drug initiation until recurrence or death. Exploratory outcomes examined associations between pre-neoadjuvant therapy biomarkers and pCR, as well as dynamic biomarker changes pre- and post-treatment in the pCR and non-pCR groups to explore neoadjuvant therapy’s immunomodulatory effects.

### Biomarker analysis

We retained specimens collected before neoadjuvant therapy and post-surgery for biomarker, immune infiltration, cell death, and pathway analysis. Preoperative tumor tissue was collected via core biopsies. Postoperative samples were obtained under the guidance of a breast pathologist, with tissue from the tumor bed collected for patients achieving pCR and residual tumor tissue collected for patients with non-pCR. sTILs were assessed according to standardized guidelines,^31^ by a single pathologist (J.H.) unaware of outcome information. RNA from pretreatment and postoperative samples underwent next-generation sequencing. We used single-sample gene set enrichment analysis (ssGSEA) to evaluate immune cell type infiltration and applied the ESTIMATE algorithm to calculate the ImmuneScore and StromalScore.^32^ These assessments are based on the proportional representation of immune and stromal cells, offering critical insights into the overall composition and purity of the tumor microenvironment. Additionally, we conducted ssGSEA incorporating bioconductor gene set variation analysis and hallmark gene sets to identify pathway enrichment associated with neoadjuvant therapy response in the transcriptome. Multiplex immunofluorescent (mIF) staining was performed on samples to evaluate the expression of immune cell infiltrate biomarkers (CD8, PDL1, CD56, and FOXP3), endothelial cell marker (CD31), and epithelial cells (panCK). The above methods are detailed in the eMethod (Supplementary Fig. 5).

### Statistical analysis

We employed Simon’s minimax two-stage design with a one-sided α error of 5% and 80% power to estimate the pCR rate as the primary endpoint. We hypothesized that adding apatinib to sintilimab alongside carboplatin plus nab-paclitaxel chemotherapy would enhance the pCR rate from 43.5% (based on NeoTRIPaPDL1 results from 2019) to 66.0%.^33^ The target accrual was a minimum of 16 patients in the first stage. If confirmed responses exceeded 7 patients, 14 additional patients were enrolled in the second stage, totaling 30 patients. Allowing for a 10% dropout rate, the estimated total sample size was 34 patients.

Efficacy was assessed in both intention-to-treat and per-protocol populations, with the former encompassing all enrolled patients who underwent at least one cycle of neoadjuvant therapy, and the latter comprising those who received at least one cycle of neoadjuvant therapy, completed surgery, and had pathological evaluation without major protocol violations. Safety analysis included all enrolled patients who received at least one dose of the study medication. pCR rate, RCB rate, and ORR with 95% confidence intervals were calculated using the Clopper-Pearson method. Summary statistics were provided for baseline demographics, disease characteristics, and adverse events. The differences in continuous variables between the two groups were assessed using the Wilcoxon rank sum test. Statistical analyses were performed using R (version 4.3.3) and SPSS (version 28), with all tests being two-sided and a significance level set at *P* < 0.05.

## Supplementary information

Sigtrans Supplementary Materials Word template

Protocol

## Data Availability

The study protocol is available in Supplementary Information. The data supporting the findings of this study are available from the corresponding author upon reasonable request. Our RNA sequencing data has been uploaded to the Genome Sequence Archive (GSA) public database for peer review. The dataset ID is HRA008190, and the data sharing link is: https://ngdc.cncb.ac.cn/gsa-human/s/s0F0G7cj.
