# Supplementary material for: Neoadjuvant apatinib addition to sintilimab and carboplatin-taxane based chemotherapy in patients with early triple-negative breast cancer: the phase 2 NeoSAC trial
Source: Signal Transduct Target Ther. 2025 Feb 7;10:41. doi: 10.1038/s41392-025-02137-7 (PMC11802755; doi:10.1038/s41392-025-02137-7)
Supplement: Supplementary file 1 — Sigtrans Supplementary Materials Word template [file 41392_2025_2137_MOESM1_ESM.docx]

Supplementary Materials for

Neoadjuvant apatinib addition to sintilimab and carboplatin-taxane based chemotherapy in patients with early triple-negative breast cancer:

the phase 2 NeoSAC trial

Guoshuang Shen, Zhilin Liu, Miaozhou Wang, Yi Zhao, Xinlan Liu, Yujin Hou, Wenbiao Ma, Jingqi Han, Xiaofeng Zhou, Dengfeng Ren, Fuxing Zhao, Zitao Li, Shifen Huang, Yongzhi Chen, Yingjian He, Yan Liu, Zijun Zhu, Yongxin Li, Jinming Li, Mengting Da, Hongnan Mo, Feng Du, Liang Cui, Jing Bai, Zhen Liu, Fei Ma, Jiuda Zhao

Correspondence to: [qhdxlz@163.com](mailto:qhdxlz@163.com); [drmafei@126.com](mailto:drmafei@126.com); [jiudazhao@126.com](mailto:jiudazhao@126.com);

**This PDF file includes:**

Materials and Methods

Figures S1 to S5

Table S1

Materials and Methods

**RNA extraction and sequencing**

The RNA extraction from pretreatment and postoperative FFPE tissues utilizes the RNeasy FFPE Kit (QIAGEN). Following extraction, the concentration and total quantity of the RNA samples are determined using Qubit 4.0. The integrity of the RNA samples is assessed with the Agilent 2100 system, and sample purity is monitored using NanoDrop spectrophotometry. Targeted removal of ribosomal RNA (rRNA) is achieved using the NEBNext® rRNA Depletion Kit (Human/Mouse/Rat) (NEB). Subsequent to rRNA depletion and fragmentation, cDNA synthesis and NGS library preparation are carried out using the NEBNext® UltraⅢ II DNA Library Prep Kit for Illumina (NEB). Library concentration and fragment size distribution are evaluated using the NGS 3K Reagent Kit (PerkinElmer). Sequencing of the samples is performed as 100 bp paired-end reads on the DNBSEQ-T7RS platform.

**RNA-seq data processing**

After eliminating terminal adaptor sequences and filtering out low-quality data with fastp software (version: 0.20.0), rRNA reads are further removed by aligning clean reads against the rRNA database downloaded from NCBI using bowtie2 (version: <2.3.5.1>). Subsequently, the clean reads, devoid of known rRNA, are aligned to the reference human genome (GRCh37) utilizing STAR software (version: 2.7.6a). Transcript assembly is performed by using StringTie software (v2.0.4). RSeQC (version: 2.6.4) assessment is employed to compute a set of quality control metrics. A predefined threshold is established, requiring an rRNA rate of less than 20% and mapped reads exceeding 40 million.

**Immune infiltration evaluation**

The GSVA^1^ R package (version 1.36.3) is implemented to conduct single-sample gene set enrichment analysis (ssGSEA), focusing on the infiltration level of 15 distinct immune cell types. Marker genes specific to each immune cell type are referenced from a published article^2^, and the gene sets are provided in Table S1. The "ESTIMATE" R package is utilized to evaluate the stromal, immune, and tumor purity scores of triple-negative breast cancer (TNBC). This assessment is grounded in the proportional representation of immune and stromal cells, providing valuable insights into the overall composition and purity of the tumor microenvironment^3^.

**Gene Set Enrichment Analysis**

The baseline samples with gene expression profiles were partitioned into two groups according to the therapeutic efficacy. Count values served as input data. The R package “limma” was employed to determine Fold Change from gene expression data between the pCR and non_pCR groups. Subsequently, all log2FoldChange values were utilized as input for the “clusterProfiler” R package to perform gene set enrichment analysis (GSEA). The Hallmark gene sets were obtained from the MSigDB database (https://www.gsea msigdb.org/gsea/msigdb/human/collections.jsp#H). Terms with |NES| >1, *p*-value < 0.05, and FDR q-value < 0.1 were considered significant.

**Oxeiptosis score**

We compared the activity (assessed via GSEA scores) of the Oxeiptosis biological process, which involves marker genes PGAM5, KEAP1, AIFM1, NRF2, and AIRE, among the different groups of TNBC. The statistical significance of the results was determined using the Wilcoxon rank sum test.

**Multiple immunofluorescence staining introduction**

To identify specific cell subsets within the tumor microenvironment (TME), we employ a multiplex immunofluorescence (mIF) staining technique using the PANO 7-plex IHC kit (Cat. No. 0004100100, Panovue, Beijing, China)^4–6^. This method involves the sequential application of different primary antibodies, followed by incubation with horseradish peroxidase-conjugated secondary antibodies and tyramide signal amplification (TSA). Microwave treatment is performed on the slides after each TSA step to enhance signal intensity. Finally, nuclei are counterstained with 4',6-diamidino-2-phenylindole (DAPI, Sigma-Aldrich) following the labeling of all human antigens^4–6^. Panel contained antibodies against CD8, PD-L1, CD56, FOXP3, CD31, and PANCK.

**Imaging analysis introduction**

Fluorescent whole-slide scanning is conducted using an Olympus VS200 scanner (Olympus Germany) equipped with an Olympus UPLXAPO 20x objective lens. The fluorescence images of the entire slide are then analyzed using OuPath software.

**Statistical Analysis**

The differences in continuous variables between the two groups were assessed using the Wilcoxon rank sum test. All statistical tests were conducted as two-sided, and results with a *p*-value less than 0.05 were deemed statistically significant. All analyses and figure creation were executed using R version 4.0.3.

**References**

1. Hänzelmann, S., Castelo, R. & Guinney, J. GSVA: gene set variation analysis for microarray and RNA-seq data. *BMC Bioinformatics* **14**, 7 (2013).

2. He, Y., Jiang, Z., Chen, C. & Wang, X. Classification of triple-negative breast cancers based on Immunogenomic profiling. *J Exp Clin Cancer Res* **37**, 327 (2018).

3. Yoshihara, K. *et al.* Inferring tumour purity and stromal and immune cell admixture from expression data. *Nat Commun* **4**, 2612 (2013).

4. Sun, Y. *et al.* Single-cell landscape of the ecosystem in early-relapse hepatocellular carcinoma. *Cell* **184**, 404-421.e16 (2021).

5. Zhang, C. *et al.* Single-cell RNA sequencing reveals intrahepatic and peripheral immune characteristics related to disease phases in HBV-infected patients. *Gut* **72**, 153–167 (2023).

6. Chen, Y.-P. *et al.* Single-cell transcriptomics reveals regulators underlying immune cell diversity and immune subtypes associated with prognosis in nasopharyngeal carcinoma. *Cell Res* **30**, 1024–1042 (2020).

Supplementary Fig. 1


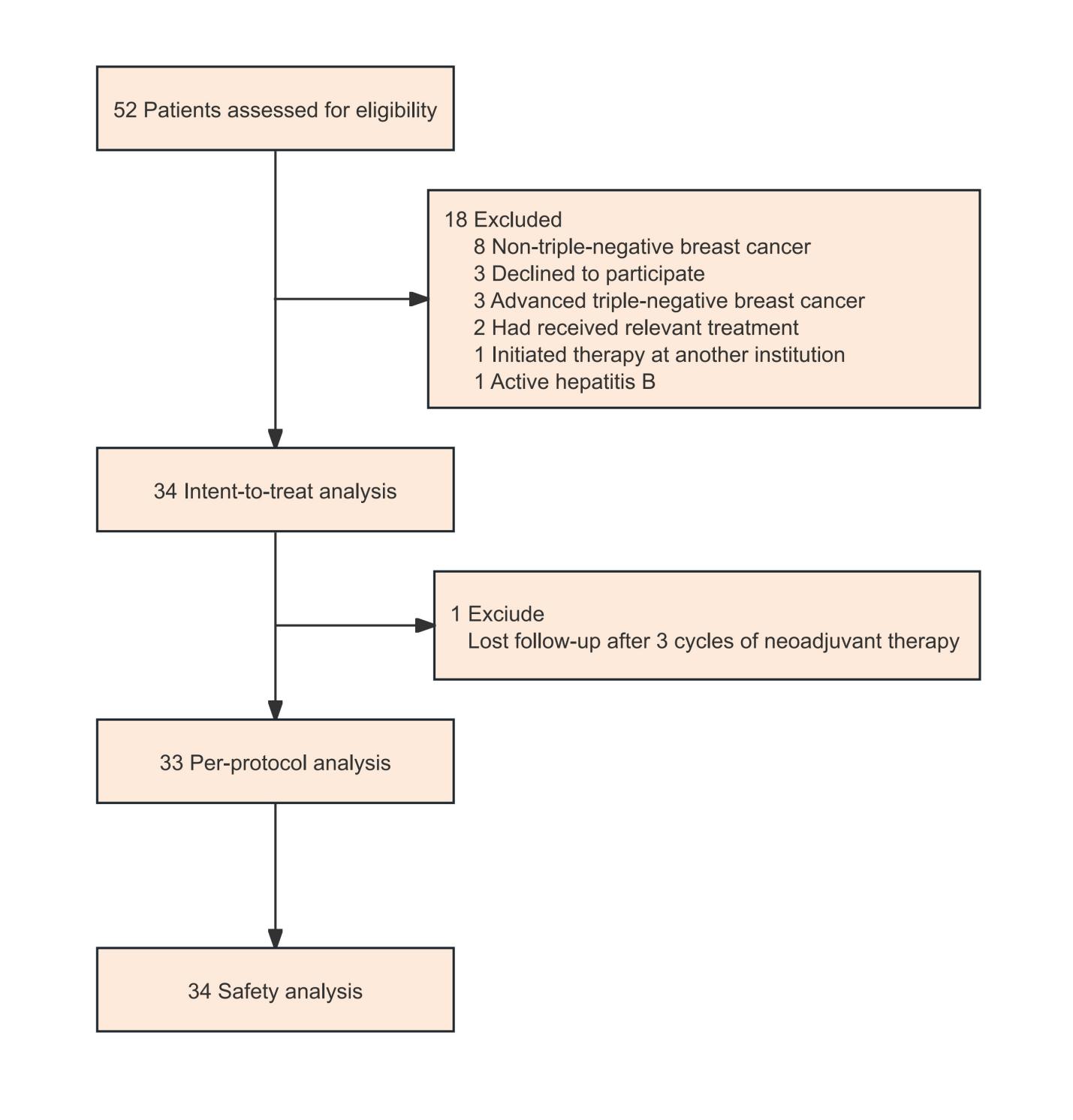


**Supplementary Fig 1. Trial profile.**

**Supplementary Fig. 2**


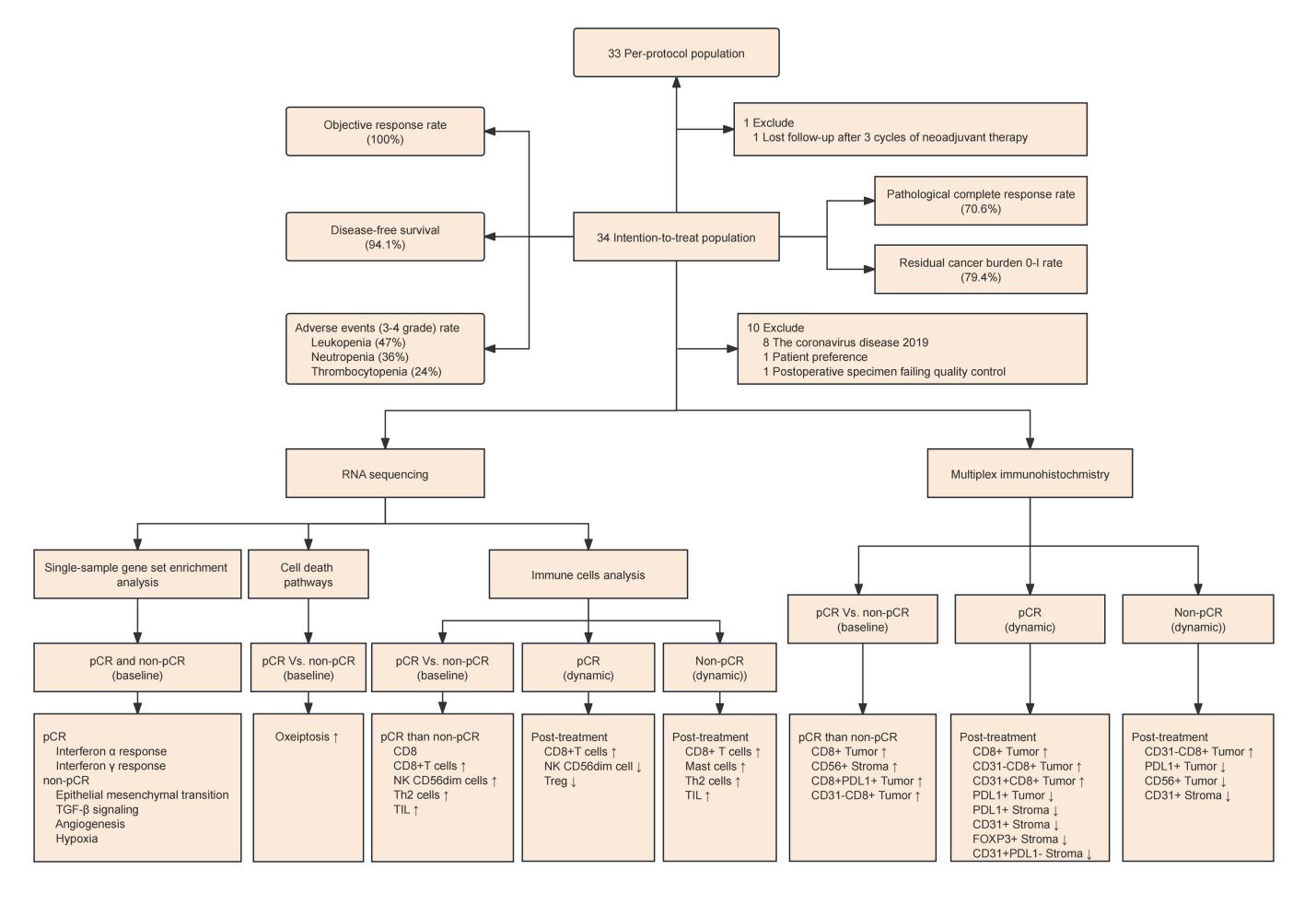


**Supplementary Fig. 2. Landscape of results in the study.**

pCR, pathological complete response

**Supplementary Fig. 3**


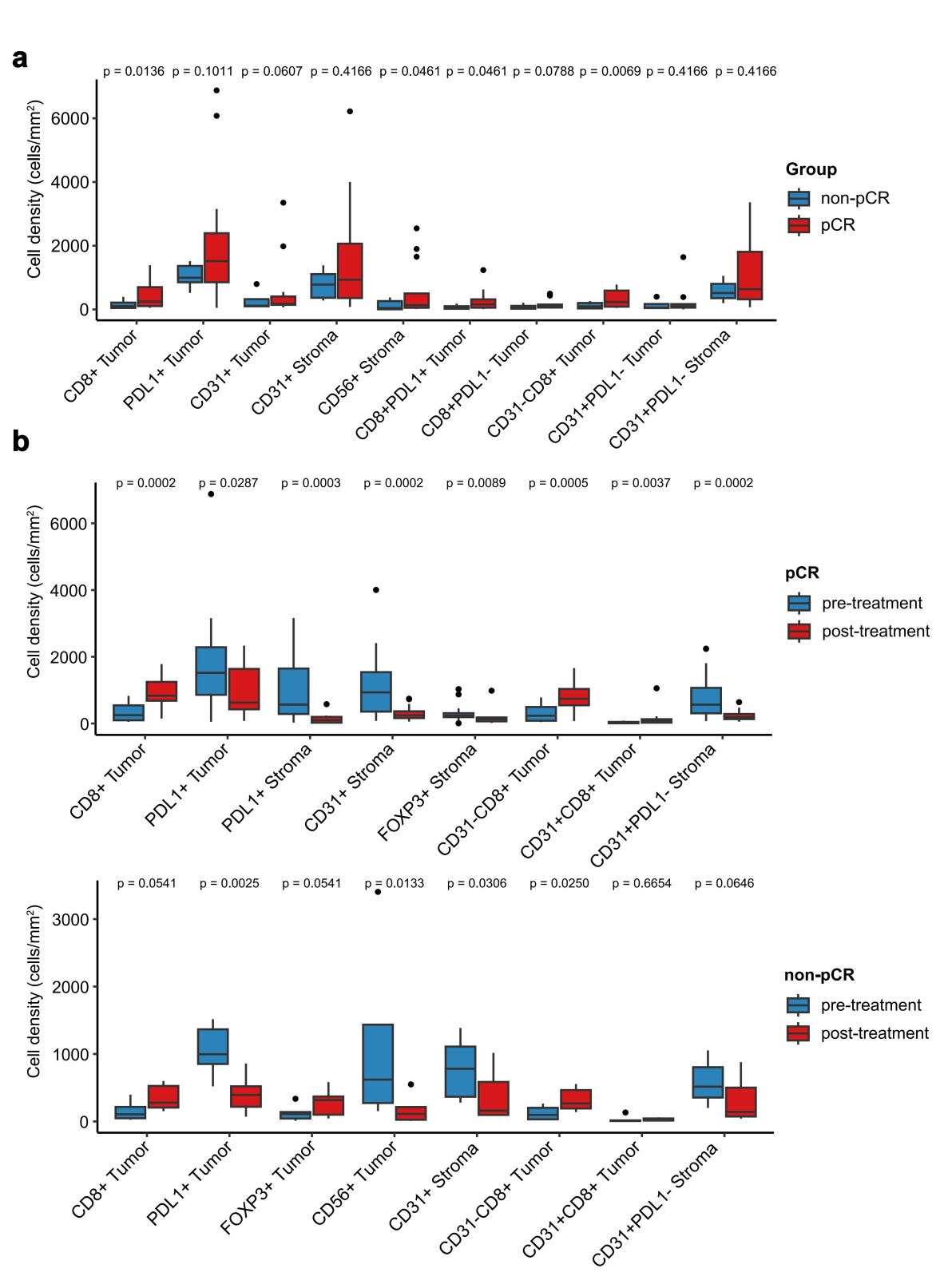


**Supplementary Fig. 3. Multiplex immunofluorescence staining of samples**

(a) Multiplex immunofluorescence staining of samples at baseline for comparison between the pCR and non-pCR groups

(b) Comparison of dynamic changes in multiplex immunofluorescence staining between the pCR and non-pCR groups pre- and post-treatment of samples

pCR, pathological complete response

**Supplementary Fig. 4**


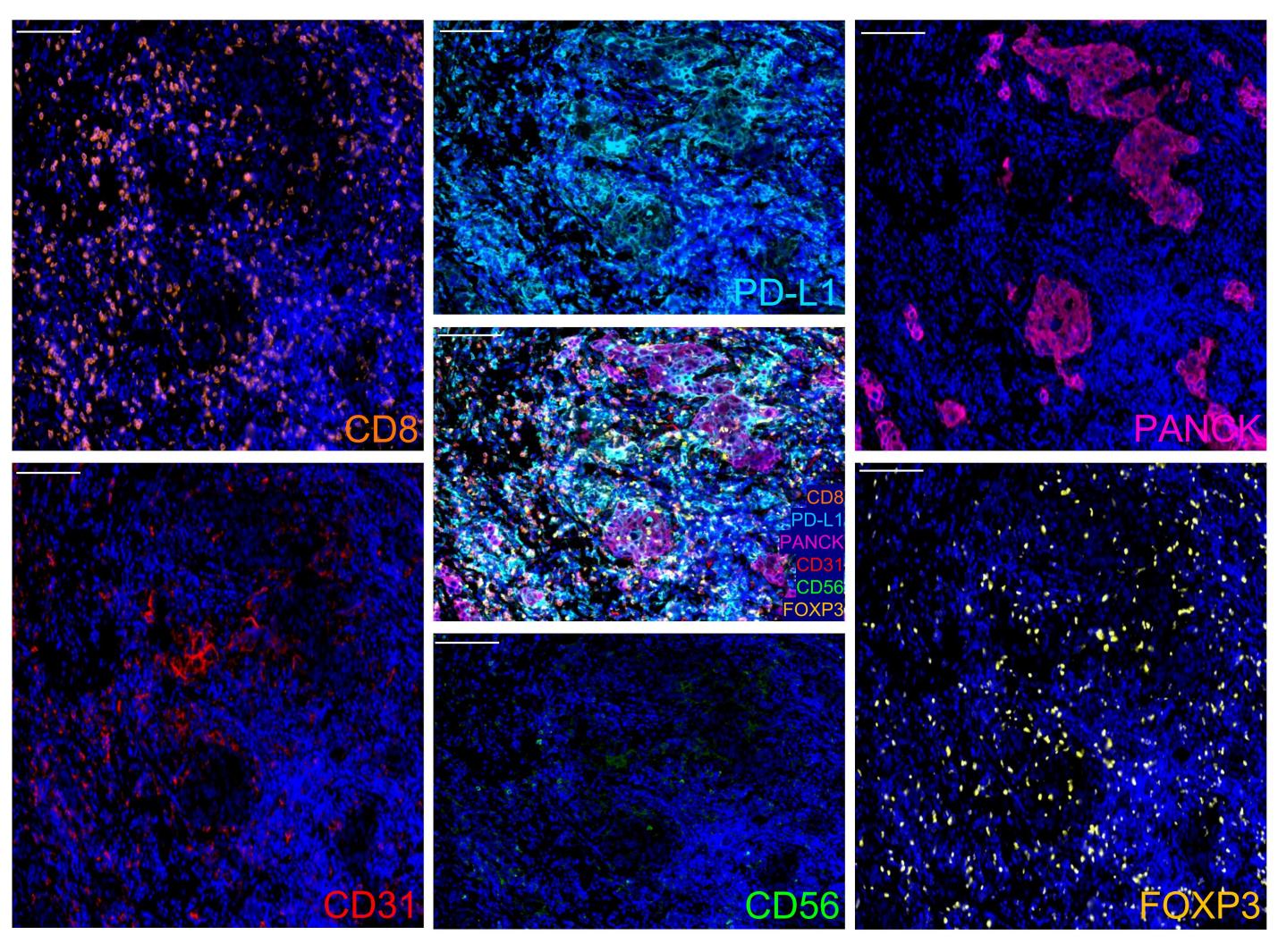


Supplementary Fig 4. Multiplex immunofluorescence staining for CD8, PD-L1, CD56, FOXP3, CD31, and PANCK. Scale bars, 100 [μ](https://baike.baidu.com/item/%CE%BC/2842656)m.

**Supplementary Fig. 5**

**
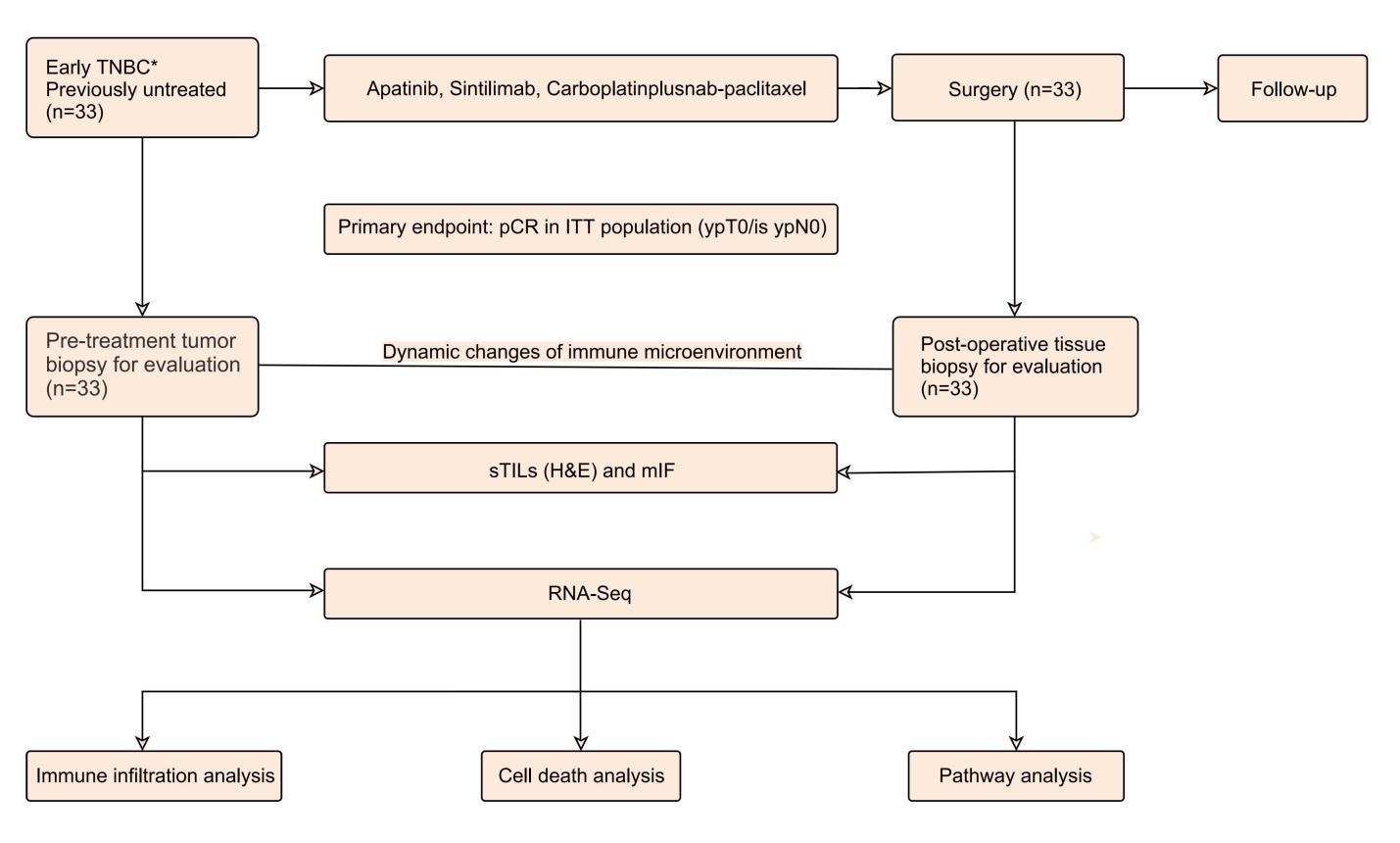
**

**Supplementary Fig. 5. NeoSAC study design and immune microenvironment assessment**

* Clinical stage II and III: T stage, T1c; N stage, N1-2. or T stage: T2-4, N stage: N0-2; TNBC: triple-negative breast cancer; pCR: pathologic complete response; ITT: Intention-to-treat; mIF: multiplex immunohistochmistry; ssGSEA: single-sample gene set enrichment analysis; PD-L1: Programmed death-ligand 1; TILs (H&E): Tumor-infiltrating lymphocytes (hematoxylin and eosin).

Supplementary Table 1

Supplementary Table. 1 Pathological and clinical responses

|  | **Intention-to-treat population (n=34)** | **Per-protocol**  **population (n=33)** * |
| --- | --- | --- |
| Total pathological complete response* | | |
| No | 9 / 34 (26.5%) | 9 / 33 (27.3%) |
| Yes | 24 / 34 (70.6%) | 24 / 33 (72.7%) |
| Residual cancer burden score* | | |
| 0 | 24 / 34 (70.6%) | 24 / 33 (72.7%) |
| I | 3 / 34 (8.8%) | 3 / 33 (9.1%) |
| II | 5 / 34 (14.7%) | 5 / 33 (15.2%) |
| III | 1 / 34 (2.9%) | 1 / 33 (3.0%) |
| Clinical responses | | |
| Complete response | 21 / 34 (61.8%) | 21 / 33 (63.6%) |
| Partial response | 13 / 34 (38.2%) | 12 / 33 (36.4%) |

Data are n (%).

*One patient in the Intention-to-treat group lost follow-up after 3 cycles of neoadjuvant therapy.
