# Supplementary material for: Neoadjuvant apatinib addition to sintilimab and carboplatin-taxane based chemotherapy in patients with early triple-negative breast cancer: the phase 2 NeoSAC trial
Source: Signal Transduct Target Ther. 2025 Feb 7;10:41. doi: 10.1038/s41392-025-02137-7 (PMC11802755; doi:10.1038/s41392-025-02137-7)
Supplement: Supplementary file 2 — Protocol [file 41392_2025_2137_MOESM2_ESM.docx]

**Neoadjuvant Apatinib Combined with Sintilimab and Carboplatin - Taxane Based Chemotherapy in Patients with Early Triple-Negative**

**Breast Cancer : A Single-Arm phase II Trial Study protocol**

**Study institute:**

**Affiliated Hospital of Qinghai University**

**General Hospital of Ningxia Medical University**

**Qinghai Provincial People' s Hospital**

**Major study investigator:Jiuda Zhao**

**Clinical Trials.gov ID: NCT04722718**


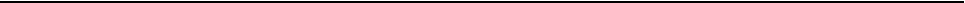


**Study protocol summary**

| Study title | Neoadjuvant Apatinib Combined with Sintilimab and Carboplatin-Taxane Based Chemotherapy in Patients with Early Triple-Negative Breast Cancer: A Single-Arm phase II Trial |
| --- | --- |
| Expected trial duration | 2 years (2021.01.01-2023.1.31) |
| Study institute | Affiliated Hospital of Qinghai University  General Hospital of Ningxia Medical University  Qinghai Provincial People's Hospital |
| PI | Jiuda Zhao |
| Rationale | Breast cancer is highly heterogeneous, with subtypes defined by estrogen receptor (ER), progesterone receptor (PR), human epidermal growth factor receptor 2 (HER-2), and Ki-67 antigen expression. These factors contribute to the classification of breast cancer into distinct subtypes, reflecting the heterogeneity of the disease. Triple negative breast cancer （ TNBC ） is one of the subtypes of breast cancer, which is defined as ≤ 1% immunohistochemistry (IHC) expression of estrogen (ER) and progesterone (PR) receptor expression and absence of human epidermal growth factor receptor 2 (HER- 2) overexpression and/or amplification according to the American Society of Clinical Oncology/College of American Pathologists (ASCO/CAP) guidelines. Clinically, TNBC is a highly invasive subtype of breast cancer, accounting for about 12%-17% of all breast cancers. Due to its aggressiveness and lack of drug targets, compared with other subtypes of breast |

|  | cancer, patients with TNBC have a shorter overall survival (OS) and the median OS of metastatic TNBC is only 8-15 months. Chemotherapy is still the main systemic treatment for TNBC, however , drug resistance develops rapidly and is poorly tolerated. Therefore, there is an urgent need to develop new treatment strategies for these patients.  To date, neoadjuvant chemotherapy (NAC) has played an important role in the treatment of early stage TNBC. Through NAC, tumor shrinkage and stage reduction can improve the surgical radical cure rate and breast conserving rate (BCR). Postoperative pathological complete response (pCR) rate is of great significance in predicting the prognosis of patients, and those who achieve pCR tend to have better prognosis. A meta-analysis of 52 studies shows that the availability of pCR for neoadjuvant therapy in patients with TNBC is significantly associated with event-free survival (EFS), with a 5-year EFS of 90% in the pCR group compared to only 57% in tumor survivors (HR=0.18). The prognosis of non-pCR or drug-resistant patients is relatively poor, and in order to reduce the recurrence and metastasis rate and improve the prognosis, intensive chemotherapy is often needed. Currently, there is no standard treatment regimen for TNBC. Previous studies have shown that the pCR rate of traditional anthracyclines followed by sequenzine therapy for TNBC can reach 25% - 40%, but there are still nearly 20% patients with recent recurrence. Therefore, the optimization of TNBC treatment regimen is still a hot spot and difficulty in current clinical studies.  In recent years, platinum drugs have attracted more and |
| --- | --- |

|  | more attention in the neoadjuvant therapy of TNBC due to their ability to significantly improve pCR rate. Carboplatin can kill cancer cells by destroying their DNA. Currently, it has become an important NAC drug in the treatment of TNBC. Studies have shown that adding the DNA damage agent carboplatin to the neoadjuvant regimen can improve the pCR rate of TNBC. In 2020, recent studies indicate that the combination of carboplatin with nab-paclitaxel demonstrates promising anti-tumor activity, boasting a high PCR rate of 48%.  In addition, antiangiogenic therapy has been considered as a potential therapeutic strategy for patients with TNBC. In 2008, bevacizumab is approved by the US Food and Drug Administration (FDA) for significantly increasing patients' progression-free survival (PFS) in combination with chemotherapy. Apatinib mesylate is developed as a new type of oral small molecule anti-angiogenesis inhibitor, mainly exerting its effects through highly selective inhibition of vascular endothelial growth factor receptor 2 (VEGFR-2) tyrosine kinase activity. It blocks vascular endothelial growth factor (VEGF) and its receptor signal transduction pathways, thus effectively inhibiting tumor angiogenesis and exhibiting anti-tumor activity. In addition, preclinical studies have shown that anti-angiogenic therapy can improve the sensitivity of anti-programmed death protein 1(PD- 1)/programmed death ligand 1(PD-L1) therapy by increasing PD-L1 expression and CD8+T cell infiltration in the tumor microenvironment. Therefore, anti-angiogenic therapy may enhance the response to PD-1/PD-L1 blockade and improve |
| --- | --- |

|  | survival.  Currently, blocking of PD-1 and PD-L1 is an attractive treatment option for TNBC, since tumor infiltrating lymphocytes (TILs) and PD-L1 are associated with favorable outcomes for TNBC. Impassion130 shows that first-line treatment with nab-paclitaxel combined with atezolizumab (anti-PD-L1 antibody) increased PFS by 2.2 months in patients with PD-L1 positive advanced TNBC, and increases OS by 7 months compared with placebo plus nab-paclitaxel. Therefore, the combination of chemotherapy and immunotherapy is proved to be effective. Meanwhile, in 2020, Impassion031 and Keynote-522 publish the results of two studies: the pCR rate after immunotherapy combined with chemotherapy neoadjuvant therapy reaches 58% and 64.8%, respectively, further confirming the high efficacy of immunotherapy combined with chemotherapy.  So far, 6 clinical trials of apatinib combined with NAC for TNBC have been registered in China. However, there are no relevant reports on immunotherapy and antiangiogenesis therapy combined with chemotherapy in the neoadjuvant treatment of TNBC at home and abroad. Based on the results of previous studies and preclinical studies, the researchers will initiate this clinical study to clarify the efficacy and safety of sintilimab and apatinib combined with chemotherapy in the neoadjuvant treatment of TNBC, to provide treatment for patients with TNBC strategy. |
| --- | --- |
| Study  objective | 1. The efficacy and safety of combining immunotherapy and antiangiogenic therapy with chemotherapy in neoadjuvant treatment for TNBC will be evaluated through the inclusion |

|  | of sintilimab and apatinib alongside NAC.  2. To clarify the breast-conserving rate (BCR), toxicity, difference in pCR rate ofpatients with PD-L1(+) and PDL1 (- ) after neoadjuvant treatment of TNBC with immunotherapy and anti-vascular therapy combined with chemotherapy and the relationship between pCR rate of immunomodulatory type (IM) and non-immunomodulatory type (non-IM) patients in” Fudan classification” .  3. RNA-seq will be performed on tissue samples from patients with TNBC enrolled in this study. The sequencing and detection results will be analyzed and integrated with bioinformatics knowledge. The anti-angiogenesis pathway and immune checkpoint inhibitor (ICI) pathway involved in this study will be combined to explore biomarkers related to the efficacy of neoadjuvant therapy, aiming to investigate tumor genome evolution and its impact on the immune microenvironment under neoadjuvant therapy. Additionally, mIF will be conducted for biomarkers analysis.  4.Through post-treatment efficacy evaluation and safety analysis, we will provide new treatment strategies for TNBC patients, increase the pCR rate of patients with TNBC, and ultimately improve the long-term survival of patients. |
| --- | --- |
| Study design | Multi-center, Prospective, Single-arm, Open-label, Phase II study |
| Methodology | II |

| Scheme | 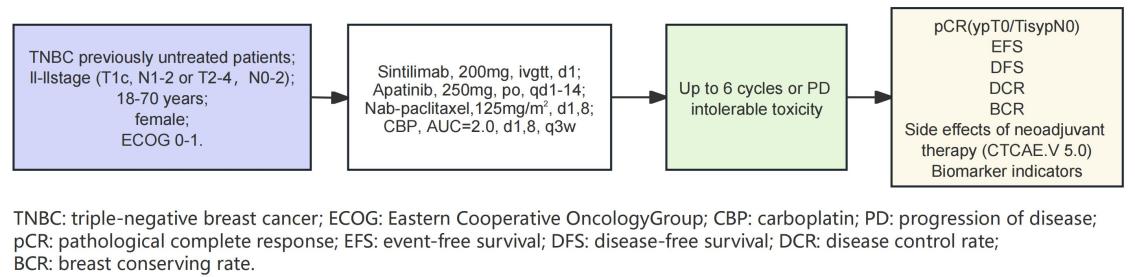 |
| --- | --- |
| Study  population | Patients with early stage TNBC |
| Number of subjects | 34 cases |
| Treatment protocol | Drug: Apatinib+Sintilimab+Nab-Paclitaxel+Carboplatin  Usage and dosage:  Apatinib: 250mg, po, d1-14days, 21days/cycle; Sintilimab: 200mg, ivgtt, d1, 21days/cycle;  Nab-Paclitaxel: 125mg/m², ivgtt, d1, d8, 21days/cycle;  Carboplatin: AUC=2.0, ivgtt, d1, d8, 21days/cycle. |
| Monitoring indicators | 1.Monitoring of blood routine,biochemical,tumor markers urine routine, blood pressure, electrocardiogram and record;  2.The patient's drug-related adverse reactions every cycle, according to the curative effect of patients;  3.Compliance and tolerance,six cycles of neoadjuvant therapy, with efficacy evaluation every two cycles (physical examination,and necessary breast ultrasound,cervical lymph node ultrasound,chest,abdominal,pelvic enhanced CT,breast enhanced MRI). |

| Inclusion Criteria | 1. The Eastern Cooperative Oncology Group (ECOG) scores ranged from 0-1 in women aged 18-70 years.  2. Pathologically confirmed patients with TNBC,which is defined as ≤ 1% immunohistochemistry (IHC) expression of estrogen (ER) and progesterone (PR) receptor expression and absence of human epidermal growth factor receptor 2 (HER-  2) overexpression and/or amplification according to the ASCO/CAP guidelines, clinical stage II and III (T stage: T1c, N stage: N1-2, or T stage: T2-4, N stage: N0-2), newly treated patients who have not received surgery or chemotherapy.  3. According to response evaluation criteria in solid tumors RECIST (version 1.1), it is confirmed by MRI or CT that at least one measurable lesion is the target lesion. If the target lesion is lymph node, the short diameter is > 1.5cm.  4. Tumor PD-L1 evaluation is confirmed through central testing of a representative tumor tissue specimen.  5. Baseline left ventricular ejection fraction (LVEF) ≥ 53% measured by echocardiogram (ECHO) or multiplegated acquisition (MUGA) scans.  6. Adequate hematologic and end-organ function.  7. Baseline laboratory tests, such as blood routine, biochemical and electrocardiogram, are normal, without chemotherapy contraindication.  8. Representative formalin-fixed, paraffin-embedded (FFPE) tumor specimen in paraffin blocks (preferred) or at least 20 unstained slides, with an associated pathology report documenting ER, PR, and HER-2 negativity.  9. For women of childbearing potential: agreement to |
| --- | --- |

|  | remain abstinent (refrain from heterosexual intercourse) or use contraceptive methods, and agreement to refrain from donating Ovum.  10. Women who are not postmenopausal or have undergone a sterilization procedure must have a negative serum pregnancy test result within 14 days prior to initiation of study drug.  11. Participant agreement to undergo appropriate surgical management including axillary lymph node surgery and partial or total mastectomy after completion of neoadjuvant  treatment. |
| --- | --- |
| Exclusion Criteria | 1. Prior history of invasive breast cancer.  2. Prior systemic therapy for treatment and prevention of breast cancer.  3. History of ductal carcinoma in situ (DCIS), except for participants treated exclusively with mastectomy > 5 years prior to diagnosis of current breast cancer.  4. History of pleomorphic lobular carcinoma in situ (LCIS), except for participants surgically managed > 5 years prior to diagnosis of current breast cancer.  5. Bilateral breast cancer.  6. Undergone incisional and/or excisional biopsy of primary tumor and/or axillary lymph nodes.  7. Axillary lymph node dissection prior to initiation of neoadjuvant therapy.  8. Have uncontrolled clinical symptoms or diseases of the heart, such as:  ( 1) heart failure above NYHA2;  (2) unstable angina pectoris; |

|  | (3) myocardial infarction occurred within 1 year;  (4) supraventricular or ventricular arrhythmia of clinical significance requires treatment or intervention.  9. Urine routine test indicates urine protein ≥ ++, or confirmed 24-hour urine protein ≥ 1.0 g.  10. Patients with hypertension (systolic blood pressure > 140mmHg, diastolic blood pressure > 90mmHg) and unsatisfactory drug control.  11. Have bleeding tendency, or combined with venous thrombosis to receive anticoagulant therapy, urine protein positive.  12. Significant abnormalities of the digestive system, such as inability to swallow, chronic diarrhea, intestinal obstruction, etc., may affect the intake, transport, or absorption of oral drugs.  13. Has major surgery within 4 weeks, or has a major traumatic injury, fracture, or poor healing wound.  14. Systemic therapy 2 years of active autoimmune disease(such as the following, but not limited to: autoimmune hepatitis, interstitial pneumonia, uveitis, enteritis, hepatitis, hypophysitis, vasculitis, nephritis , hyperthyroidism, decreased thyroid function; subjects suffering from vitiligo or asthma in childhood has been completely relieved, and adults without any intervention can be included; subjects requiring bronchodilators for medical intervention can not be included), diagnosis of immune deficiency in 1 weeks or the use of immunosuppressive therapy, the history of human immunodeficiency virus (HIV) infection, had glucocorticoid treatment history of non infectious pneumonia, suffering from |
| --- | --- |

|  | pneumonia, active tuberculosis, active hepatitis b or hepatitis c virus (HCV) infection, and is being treated for a whole body of any active infection.  15. History of severe allergic, anaphylactic, or other hypersensitivity reactions to chimeric or humanized antibodies or fusion proteins.  16. Known hypersensitivity to biopharmaceuticals produced in Chinese hamster ovary cells.  17. Known allergy or hypersensitivity to the components of the formulations of sintilimab, appatinib, albumin paclitaxel or carboplatin.  18. Prior allogeneic stem cell or solid organ transplantation.  19. Administration of a live attenuated vaccine within 4 weeks prior to initiation of study treatment or anticipation of need for such a vaccine during the study.  20. Patients who have previously received CTLA-4, Tim3, LAG3 and other antibodies or T cell costimulation therapy.  21. Any other disease, metabolic dysfunction, physical examination finding, or clinical laboratory finding giving reasonable suspicion of a disease or condition that contraindicates the use of an investigational drug or that may affect the interpretation of the results or render the participant at high risk from treatment complications.  22. History of cerebrovascular accident within 12 months.  23. Pregnant or lactating, or intending to become pregnant during the study.  24. Patients diagnosed with inflammatory breast cancer.  25. Patients with a second primary tumor at the same time.  26. The doctor considers that the patient is not suitable for |
| --- | --- |

|  | enrollment. |
| --- | --- |
| The primary endpoint | Pathological complete response (pCR) rate  pCR: using the definition of ypT0/TisypN0 (namely:no invasive residual in breast or nodes; noninvasive breast residuals allowed) at the time of definitive surgery according to American Joint Committee on Cancer (AJCC) Staging System.  [Time Frame: 4-6 months] |
| The  secondary endpoints | 1. EFS (event-free survival)  12  EFS defined as the duration from initiation to the occurrence of disease progression preventing definitive surgery, local or distant recurrence, the development of a second primary cancer, or death from any cause, whichever come first, is assessed by an investigator blinded to the trial- group assignments.  [Time Frame: 2 years]  2. DFS (disease-free survival)  DFS defined as the time from drug onset until recurrence or death for various reasons.  [Time Frame: 2 years]  3. DCR（disease control rate）  CR (complete response)+PR (partial response)+SD (stable disease)  [Time Frame: 4 months]  4. BCR (breast conserving rate)  [Time Frame: 4 months] 5. Rate of BpCR and RCB  The pCR rate of breast and the overall RCB rate. [Time Frame: 4-6 months] |

|  | 6. Side effects of neoadjuvant therapy (CTCAE.V5.0) [Time Frame: 4 months] |
| --- | --- |
| Exploratory endpoints | The exploratory outcomes focus on examining the associations between pre-neoadjuvant therapy biomarkers and pCR, as well as the dynamic changes of biomarkers before and after treatment in both the pCR and non-pCR groups. This analysis aims to elucidate the immunomodulatory effects of neoadjuvant therapy. |
| Baseline data | 1. Basic patient information (including patient code/number in registration database, demographic information).  2. Detailed medical history is collected to clarify the pathological diagnosis of tumors, understand the past treatment history and efficacy, and the current medication status of subjects.  3. Laboratory tests: blood routine, urine routine, serum biochemistry, tumor markers, thyroid function, cardiac function (BNP, etc.) and virology and other tests when  necessary.  4. Electrocardiogram and/or echocardiography, breast and superficial lymph node ultrasound, breast X-ray, breast- enhanced MRI, chest and abdominal CT, head MRI, bone scan, PET-CT if deemed necessary.  5. Functional capacity evaluation. |
| Statistical method | The statistical analysis of all data will be conducted using SPSS software (IBM, Armonk, NY, USA; Version 28.0) and R. Statistical description of all efficacy indicators obtained at each observation time point. In general, continuous variables will be statistically described using case number, mean value, standard deviation, quartile, median, minimum value, and |

|  | maximum value. Classification variables will be statistically described using the frequency and percentage of each category. The Kaplan-Meier method will be used to estimate EFS and DFS. Additionally, the Kaplan-Meier method and log-rank test will be employed for univariate identification of EFS and DFS based on demographic data, baseline clinical information, and toxicity analysis. Moreover, the Cox regression model will be utilized to study the combined effects of these variables on EFS and DFS in multivariate analysis. The therapeutic effectiveness will be tested using Fisher's exact test. *P <* 0.05 indicates statistical significance. |
| --- | --- |
| Sample size calculation | We use a Simon’s minimax two-stage design with a one- sided α error of 5% and a power of 80% to estimate the pCR rate as the primary endpoint. We hypothesize that the addition of apatinib to sintilimab combined with carboplatin plus nab- paclitaxel chemotherapy improves the pCR rate from 43.5% (based on the NeoTRIPaPDL1 results reported in 2019) to 66.0%. The target accrual is a minimum of 16 patients in the first stage, and if responses are confirmed in more than 7 patients, 14 additional patients will be accrued in the second stage, with a total of 30 patients. Assuming a 10% drop-out rate, we estimate a total sample size of 34 patients. |
| Following-up data | 1. Treatment information at follow-up visit.  2. Evaluation of clinical efficacy (CR, PR, SD, PD).  3. Safety capability evaluation.  4. Laboratory examination.  5. Whether the treatment plan is adjusted and the treatment plan after adjustment. |

| Follow-up  frequency and the cycle | All subjects undergo objective efficacy evaluation (RECIST.v1.1) and safety evaluation (CTCAE.v5.0) every 2 cycles according to solid tumor efficacy evaluation criteria. |
| --- | --- |
| Follow-up personnel | Research assistant or research nurse |
| Follow-up way | Outpatient or inpatient examination, email contact, telephone contact, email contact, or other online contact to obtain follow- up data;  Follow-up data may be provided by the patient himself or, when not available, by a relative of the patient. |


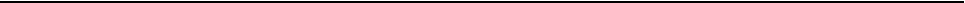


**Abbreviation**

| ER | Estrogen Receptor |
| --- | --- |
| PR | Progesterone Receptor |
| HER-2 | Human epidermal growth factor receptor-2 |
| TNBC | Triple-negative Breast Cancer |
| IHC | Immunohistochemistry |
| NAC | Neoadjuvant Chemotherapy |
| ICI | Immune Checkpoint Inhibitor |
| OS | Overall Survival |
| EFS | Event-Free Survival |
| DFS | Disease-free survival |
| PFS | Progression-Free Survival |
| DCR | Disease Control Rate |
| LAR | Luminal Androgen Receptor |
| IM | Immunomodulatory |
| BLIS | Basal-like and Immune-Suppressed |
| MES | Mesenchymal-like |
| PCR | Pathological Complete Response |
| BPCR | Breast Pathological Complete Response |
| RCB | Residual cancer burden |
| FDA | Food and Drug Administration |
| VEGFR-2 | Vascular Endothelial Growth Factor Receptor-2 |
| PD-1 | Programmed Death Protein 1 |


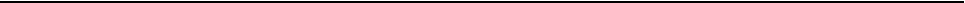


| PD-L1 | Programmed Death Ligand 1 |
| --- | --- |
| TILS | Tumor Infiltrating Lymphocytes |
| WES | Whole-Exome Sequencing |
| RNA-seq | RNA Sequencing |
| mIF | multiple immunofluorescence |
| ECOG | The Eastern Cooperative Oncology Group |
| LVEF | Left Ventricular Ejection Fraction |
| ECHO | Echocardiogram |
| DCIS | Ductal Carcinoma in situ |
| LCIS | Lobular Carcinoma in situ |
| AJCC | American Joint Committee on Cancer |
| MP | Miller&Payne |
| CR | Complete Response |
| PR | Partial Response |
| SD | Stable Disease |
| PD | Progressive Disease |
| TMB | Tumor Mutation Burden |
| AST | Aspartate Aminotransferase |
| ALT | Alanine Aminotransferase |
| AE | Adverse Event |
| SAE | Severe Adverse Event |
| ADRs | Adverse Drug Reactions |


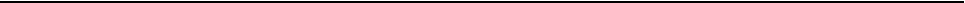


| SAP | Statistical Analysis Plan |
| --- | --- |
| CBR | Breast Preservation Rate |
| SOC | Organ System Category |
| EC | Ethics Committee |
| IRB | Institutional Review Board |
| GCP | Good Clinical Practice |

**1. Research background**

In the past decades, the incidence of breast cancer in humans has continued to rise, posing a huge threat to the lives and health of patients worldwide. According to statistics released by the American Cancer Society, in 2019, there are more than 271,000 new cases of breast cancer and about 42,260 deaths in the United States[ 1] . Breast cancer, as a highly heterogeneous disease, can be classified based on the expression of estrogen receptor (ER), progesterone receptor (PR), human epidermal growth factor receptor 2 (HER- 2), and the Ki-67 antigen, which is divided into several subtypes[2] . Triple negative breast cancer （ TNBC ） is one of the subtypes of breast cancer, which is characterized by the lack ofprotein expression of ER, PR and HER- 2[3-4] . Clinically, TNBC is a very aggressive subtype of breast cancer, accounting for about 12%-17% of all breast cancers[5-6] . Due to its aggressiveness and lack of drug targets, compared with other subtypes of breast cancer, patients with TNBC have a shorter overall survival (OS) and the median OS of metastatic TNBC is only 8-15 months. Chemotherapy is still the main systemic treatment for TNBC, but drug resistance develops rapidly and is poorly tolerated. Therefore, there is an urgent need to develop new treatment strategies for these patients[7-8] .

The clinical and molecular heterogeneity of TNBC is now well understood. Gene expression analysis shows that immune markers, androgen receptors, mesenchymal phenotypes, stem cell markers and basic markers are all related to TNBC subtypes[9] . With reference to previous studies, according to the results of transcriptomics research, and based on transcriptome data, China's "Fudan Classification" divides TNBC into 4 subtypes: ( 1) luminal androgen receptor (LAR), (2) immunomodulatory (IM), (3) basal-like and immune- suppressed (BLIS) and (4) mesenchymal-like (MES). Possible therapeutic

targets or biomarkers have been identified for each subtype[ 10] .


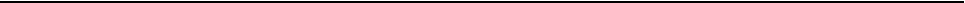
To date, neoadjuvant chemotherapy (NAC) has played an important role in

the treatment of locally advanced TNBC patients. Through NAC, tumor shrinkage and stage reduction can improve the surgical radical cure rate and breast conserving rate (BCR). Postoperative pCR rate is of great significance in predicting the prognosis of patients, and those who achieve pCR tend to have better prognosis. A meta-analysis of 52 studies shows that the availability of pCR for neoadjuvant therapy in Patients with TNBC is significantly associated with event-free survival (EFS), with a 5-year EFS of 90% in the pCR group and only 57% in tumor survivors (HR=0.18)[ 11] . The prognosis of non-pCR or drug-resistant patients is relatively poor, and in order to reduce the recurrence and metastasis rate and improve the prognosis, intensive chemotherapy is often needed. Currently, there is no standard treatment regimen for TNBC. Previous studies have shown that the pCR rate of traditional anthracyclines followed by sequenzine therapy for TNBC can reach 25%-40%, but there are still nearly 20% patients with recent recurrence[ 12-14] . Therefore, the optimization of TNBC treatment regimen is still a hot spot and difficulty in current clinical studies.

In recent years, platinum drugs have attracted more and more attention in the neoadjuvant therapy of TNBC due to their ability to significantly improve pCR rate. Carboplatin can kill cancer cells by destroying their DNA. Currently, it has become an important NAC drug in the treatment of TNBC. Studies have shown that adding the DNA damage agent carboplatin to the neoadjuvant regimen can improve the pCR rate of TNBC[ 15-16] . In 2020, recent studies indicate that the combination of carboplatin with nab-paclitaxel demonstrates promising anti-tumor activity, boasting a high pathological complete response (pCR) rate of48%[ 17] .

In addition, antiangiogenic therapy has been considered as a potential therapeutic strategy for patients with TNBC. In 2008, bevacizumab is approved by the US Food and Drug Administration (FDA) for significantly increasing patients' PFS in combination with chemotherapy. Apatinib


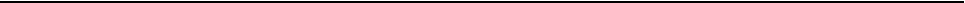
mesylate is developed as a new type of oral small molecule anti-angiogenesis

inhibitor, mainly exerting its effects through highly selective inhibition of VEGFR-2 tyrosine kinase activity. It blocks VEGF and its receptor signal transduction pathways, thus effectively inhibiting tumor angiogenesis and exhibiting anti-tumor activity[ 18-19] . In addition, preclinical studies have shown that anti-angiogenic therapy can improve the sensitivity of anti- programmed death protein 1(PD-1)/programmed death ligand 1(PD-L1) therapy by increasing PD-L1 expression and CD8+ T cell infiltration in the tumor microenvironment. Therefore, anti-angiogenic therapy may enhance the response to PD-1/PD-L1 blockade and improve survival[20] .

Currently, blocking of PD-1 and PD-L1 is an attractive treatment option for TNBC, since tumor infiltrating lymphocytes (TILs) and PD-L1 are associated with favorable outcomes for TNBC. Impassion130 shows that first-line treatment with nab-paclitaxel combined with atezolizumab (anti-PD-L1 antibody) increased PFS by 2.2 months in patients with PD-L1 positive advanced TNBC, and increased OS by 7 months compared with placebo plus nab-paclitaxel. Therefore, the combination of chemotherapy and immunotherapy is proved to be effective[21] . Meanwhile, in 2020, Impassion031[22] and Keynote-522[23] published the results of two studies: the pCR rate after immunotherapy combined with chemotherapy neoadjuvant therapy reached 58% and 64.8%, respectively, further confirming the high efficacy of immunotherapy combined with chemotherapy.

TNBC as one of a kind of high heterogeneity of breast cancer subtypes, its aggressive is strong, postoperative recurrence occurred more transfer, lack of effective treatment of molecular therapeutic targets, endocrine therapy and anti HER2 therapy often invalid, therefore, compared with other subtypes of breast cancer, TNBC after NAC to pCR has more significant prognostic value. Currently, there is no standard treatment for TNBC except traditional anthracycline and taxane chemotherapy regimens. Therefore, it is critical to optimize the NAC regimens for TNBC to increase the pCR rate and improve


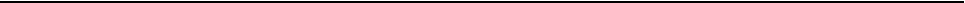
the long-term prognosis ofpatients.

So far, 6 clinical trials of apatinib combined with NAC for TNBC have been registered in China. However, there are no relevant reports on immunotherapy and antiangiogenesis therapy combined with chemotherapy in the neoadjuvant treatment of TNBC at home and abroad. Based on the results of previous studies and preclinical studies, the researchers will initiate this clinical study to clarify the efficacy and safety of sintilimab and apatinib combined with chemotherapy in the neoadjuvant treatment of TNBC, in order to provide treatment for patients with TNBC strategy.

**2. Research Purpose**

**2.1 The main purpose of this study is:**

Through the addition of Sintilimab and Apatinib in NAC, we will explore the efficacy and safety of combined immunotherapy and antiangiogenic therapy in neoadjuvant therapy of TNBC.

**2.2 The following data can be obtained from this study：**

Efficacy and safety of immunotherapy and antivascular therapy combined with chemotherapy in neoadjuvant therapy for TNBC.

We will analyze biomarkers predictive of response to neoadjuvant treatment in pre-neoadjuvant therapy specimens, as well as differences in the dynamic changes of biomarkers before and after treatment between pCR and no pCR in paired pre- and post-neoadjuvant therapy specimens, in order to clarify the role of neoadjuvant therapy at the cellular and molecular levels.

Toxicity, BCR, difference in pCR rate of patients with PD-L1 (+) and PD- L1 (-), the relationship between pCR rate of IM and non-IM type, and the relationship between TMB and pCR rate of patients after TNBC neoadjuvant therapy.Pathological response assessed by the MP scoring system combined


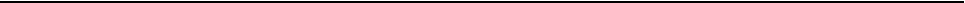
with residual disease in lymph nodes after neoadjuvant therapy. The

correlation between genomic analyses and pCR.The correlation between genomic analyses and pCR. The correlation between TILs and pCR. Residual cancer burden (RCB) will also be used for pathological evaluation.

Meanwhile, RNA-seq will be performed on tissue samples ofpatients with TNBC enrolled in this study. The sequencing and detection results will be analyzed and integrated with bioinformatics knowledge, and the anti- angiogenesis pathway and ICI pathway involved in this study will be combined to explore biomarkers related to the efficacy of neoadjuvant therapy. Multiplex immunofluorescence (mIF) will also be used for biomarkers detection. To explore the tumor genome evolution and its effect on immune microenvironment under neoadjuvant therapy.

**3. Inclusion and Exclusion Criteria**

**3.1 Inclusion Criteria：**

1. The Eastern Cooperative Oncology Group (ECOG) scores ranged from 0-1 in women aged 18-70 years.

2. Pathologically confirmed patients with TNBC.clinical stage II and III (T stage:T1c, N stage: N1-2, or T stage: T2-4, N stage: N0-2),newly treated patients who have not received surgery or chemotherapy.

3. According to response evaluation criteria in solid tumors RECIST (version 1.1), it is confirmed by MRI or CT that at least one measurable lesion is the target lesion. If the target lesion is lymph node, the short diameter is > 1.5cm.

4. Confirmed tumor PD-L1 evaluation as documented through central testing of a representative tumor tissue specimen.

5. Baseline left ventricular ejection fraction (LVEF) ≥ 53% measured by echocardiogram (ECHO) or multiplegated acquisition (MUGA) scans.

6. Adequate hematologic and end-organ function.

7. Baseline laboratory tests, such as blood routine, biochemical and


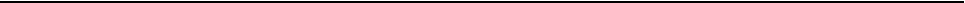
electrocardiogram, are normal, without chemotherapy contraindication.

8. Representative formalin-fixed, paraffin-embedded (FFPE) tumor specimen in paraffin blocks (preferred) or at least 20 unstained slides,with an associated pathology report documenting ER, PR, and HER-2 negativity.

9. For women of childbearing potential: agreement to remain abstinent (refrain from heterosexual intercourse) or use contraceptive methods,and agreement to refrain from donating ovums.

10. Women who are not postmenopausal or have undergone a sterilization procedure must have a negative serum pregnancy test result within 14 days prior to initiation of study drug.

11. Participant agreement to undergo appropriate surgical management including axillary lymph node surgery and partial or total mastectomy after completion of neoadjuvant treatment.

**3.2 Exclusion Criteria：**

1. Prior history of invasive breast cancer.

2. Prior systemic therapy for treatment and prevention of breast cancer.

3. History of ductal carcinoma in situ (DCIS), except for participants treated exclusively with mastectomy > 5 years prior to diagnosis of current breast

cancer.

4. History of pleomorphic lobular carcinoma in situ (LCIS), except for participants surgically managed > 5 years prior to diagnosis of current breast

cancer.

5. Bilateral breast cancer.

6. Undergone incisional and/or excisional biopsy of primary tumor and/or axillary lymph nodes.

7. Axillary lymph node dissection prior to initiation of neoadjuvant therapy.

8. Have uncontrolled clinical symptoms or diseases of the heart, such as:

( 1) heart failure above NYHA 2

(2) unstable angina pectoris


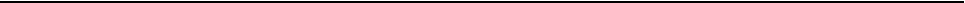
(3) myocardial infarction occurred within 1 year.

(4) supraventricular or ventricular arrhythmia of clinical significance requires treatment or intervention.

9. Urine routine test indicates urine protein ≥ ++, or confirmed 24-hour urine protein ≥ 1.0 g.

10. Patients with hypertension (systolic blood pressure > 140mmHg,diastolic blood pressure > 90mmHg) and unsatisfactory drug control.

11. Have bleeding tendency, or combined with venous thrombosis to receive anticoagulant therapy, urine protein positive.

12. Significant abnormalities of the digestive system, such as inability to swallow, chronic diarrhea, intestinal obstruction, etc., may affect the intake, transport, or absorption of oral drugs.

13. Has major surgery within 4 weeks, or has a major traumatic injury, fracture, or poor healing wound.

14. Systemic therapy 2 years of active autoimmune disease(such as the following, but not limited to: autoimmune hepatitis, interstitial pneumonia, uveitis, enteritis, hepatitis, hypophysitis, vasculitis, nephritis , hyperthyroidism, decreased thyroid function; subjects suffering from vitiligo or asthma in childhood has been completely relieved, and adults without any intervention can be included; subjects requiring bronchodilators for medical intervention can not be included), diagnosis of immune deficiency in 1 weeks or the use of immunosuppressive therapy, the history of human immunodeficiency virus (HIV) infection, had glucocorticoid treatment history of non infectious pneumonia, suffering from pneumonia, active tuberculosis, active hepatitis b or hepatitis c virus (HCV) infection, and is being treated for a whole body of any active infection.

15. History of severe allergic, anaphylactic, or other hypersensitivity reactions to chimeric or humanized antibodies or fusion proteins.

16. Known hypersensitivity to biopharmaceuticals produced in Chinese hamster ovary cells.


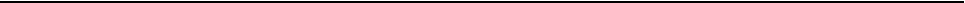
17. Known allergy or hypersensitivity to the components of the formulations of sintilimab, appatinib,albumin paclitaxel or carboplatin.

18. Prior allogeneic stem cell or solid organ transplantation.

19. Administration of a live attenuated vaccine within 4 weeks prior to initiation of study treatment or anticipation of need for such a vaccine during the study.

20. Patients who have previously received CTLA-4, Tim3, LAG3 and other antibodies or T cell costimulation therapy.

21. Any other disease, metabolic dysfunction, physical examination finding, or clinical laboratory finding giving reasonable suspicion of a disease or condition that contraindicates the use of an investigational drug or that may affect the interpretation of the results or render the participant at high risk from treatment complications.

22. History of cerebrovascular accident within 12 months.

23. Pregnant or lactating, or intending to become pregnant during the study.

24. Patients diagnosed with inflammatory breast cancer.

25. Patients with a second primary tumor at the same time.

26. The doctor considers that the patient is not suitable for enrollment.

**4. Research proposal**

**4.1 Study Design Brief:**

Type of study: multicenter, prospective, single-arm, open-label, phase II study.

**4.2 Therapeutic regimen and dosage**

Apatinib+Sintilimab+Nab-Paclitaxel+Carboplatin Apatinib: 250mg po, d 1-14 days, 21days/cycle; Sintilimab: 200mg, ivgtt, d 1,21days/cycle;

Nab-Paclitaxel: 125mg/m2, ivgtt, d 1, d8, 21days/cycle; Carboplatin: AUC= 2, ivgtt, d 1, d8, 21days/cycle.

**4.3 Medication reduction and withdrawal plan**

**Table S4. Recommended thera eutic ad ustment of Sintilimab**

**p j**

| Immune  related adverse  events | Severity Degree | Treatment adjustment |
| --- | --- | --- |
| pneumonia | Grade 2 | Suspend the administration  until the adverse reaction  recovers to Grade 0-1 |
|  | Grade 3 or 4 or Repetitive Grade 2 | Permanent drug withdrawal |
| Diarrhea and colitis | Grade 2 or 3 | Suspend the administration  until the adverse reaction  recovers to Grade 0-1 |
|  | Grade 4 | Permanent drug withdrawal |
| Hepatitis  (applicable to  the  hepatocellular  carcinoma  patients) | Grade 2, Aspartate aminotransferase (AST) and alanine aminotransferase (ALT) in 3  ~  5 times the upper limit of normal value (ULN) or total bilirubin (TBIL) in 1.5  ~  3ULN | Suspend the administration  until the adverse reaction  recovers to Grade 0-1 |
|  | Grade 3 or 4, AST and ALT > 5ULN, or TBIL > 3ULN | Permanent drug withdrawal |
| Nephritis | Grade 2 or 3 elevated serum creatinine | Suspend the administration  until the adverse reaction  recovers to Grade 0-1 |
|  | Grade 4 elevated serum creatinine | Permanent drug withdrawal |
| Endocrine disease | Symptomatic grade 2 or 3 hypothyroidism;  Grade 2 or 3 hyperthyroidism； Grade 2 or 3 hypophysitis；  Grade 2 adrenal insufficiency；  Grade 3 hyperglycemia or type 1 diabetes; | Suspend the administration  until the adverse reaction  recovers to Grade 0-1 |
|  | Grade 4 hypothyroidism;  Grade 4 hyperthyroidism;  Grade 4 hypophysitis;  Grade 3 or 4 adrenal insufficiency;  Grade 4 hyperglycemia or type 1 diabetes; | Permanent drug withdrawal |
| Adverse skin reaction | Grade 3 | Suspend the administration  until the adverse reaction  recovers to Grade 0-1 |
|  | Grade 4, Stevens Johnson syndrome (SJS)  or toxic epidermal necrosis loose solution  (TEN) | Permanent drug withdrawal |
| Thrombocytop enia | Grade 3 | Suspend the administration  until the adverse reaction  recovers to Grade 0-1 |

|  | Grade 4 | Permanent drug withdrawal |
| --- | --- | --- |
| Other immune related adverse  events | Grade 3 or 4 blood amylase increase or  lipase increase;  Grade 2 or 3 pancreatitis;  Grade 2 myocarditis;  Grade 2 or 3 other immune related adverse  reactions occurred for the first time; | Suspend the administration  until the adverse reaction  recovers to Grade 0-1 |
|  | Grade 4 pancreatitis or any grade of  recurrent pancreatitis;  Grade 3 or 4 myocarditis;  Grade 3 or 4 encephalitis;  Grade 4 other immune related adverse  reactions occurred for the first time; | Permanent drug withdrawal |
| Recurrent or  persistent  adverse events | Recurrent grade 3 or 4 (except endocrine  disease);  Grade 2 or 3 adverse reactions did not  improve to Grade 0-1 within 12 weeks after  the last administration (except endocrine  diseases);  Corticosteroids failed to reduce to ≤ 10  mg/day prednisone equivalent dose within  12 weeks after the last administration; | Permanent drug withdrawal |
| Infusion reaction | Grade 2 | Reduce the infusion rate or  suspend the administration.  When the symptoms are  relieved, consider resuming  the administration and  closely observe |
|  | Grade 3 or 4 | Permanent drug withdrawal |

**4.3.2 Recommended therapeutic adjustment of Apatinib(Table S5)**

**Table S5. Recommended thera eutic ad ustment of A atinib**

**p j p**

| Classification of adverse events | NCI grade | Regulation of dose adjustment |
| --- | --- | --- |
| Hematology adverse event | Grade 3 | The drug will be suspended, and the drug will be  continued at the original dose after the adverse  reaction has recovered to ≤ grade 2. If grade 3 or  above adverse reactions occur again, the drug will be  continued after decreasing one dose. |
|  | Grade 4 | Suspend medication, and continue to use the drug after  the adverse reaction recovers to ≤ level 2 and one dose  is lowered (125mg). |

| Non hematologic  adverse events  (Hypertension;  Proteinuria;  Vomiting and  Diarrhea;  Hyperbilirubinemia,  AST↑, ALT↑;  Anorexia;  Palmoplantar  redness syndrome;  Fatigue;  Headache,  dizziness, loss of  sensation;  Dysphonia, pharyngitis；  Bradyarrhythmia;  Back&limb pain;  urinary tract  infection;  Insomnia;  Tinnitus and  vertigo;  Breast pain;  Hypersensitivity;  Occult blood  positive) | Grade 3 | The drug will be suspended, and it will be continued at  the original dose after the adverse reaction has  recovered to ≤ grade 2. If grade 3 or above adverse  reactions occur again, the drug will be continued after  decreasing one dose. |
| --- | --- | --- |
|  | Grade 4 | Suspend medication, and continue to use the drug after  the adverse reaction recovers to ≤ level 2 and one dose  is lowered (125mg). |

Note: The above evaluation is conducted using the NCI-CTCAE5.0 scale of common drug toxicity as designated by the National Cancer Institute.

**4.4 The implementation checklist for the NeoSAC research is as follows(Table S6):**

**Table S6. Stud the checklist of im lementation rocess**

**y p p**

|  | Screening  and  enrollment | First  medication | During the  period of drug delivery (21 days/cycle,  once /2 cycles) | Data  Auditing | statistic analysis |
| --- | --- | --- | --- | --- | --- |
| Patient registration screening | √ |  |  |  |  |

| Informed consent | √ |  |  |  |  |
| --- | --- | --- | --- | --- | --- |
| Baseline data collection | √ |  |  |  |  |
| First visit data collection |  | √ |  |  |  |
| Inpatient/outpat  ient treatment data collection |  | √ |  |  |  |
| Therapeutic  effect  evaluation |  |  | √ |  |  |
| Safety  capability evaluation |  |  | √ |  |  |
| Data review and data query  answering |  |  |  | √ |  |
| Statistical  analysis and  statistical report  writing |  |  |  |  | √ |

**4.5 The simplified flowchart of the NeoSAC research is as follows(Figure S6):**


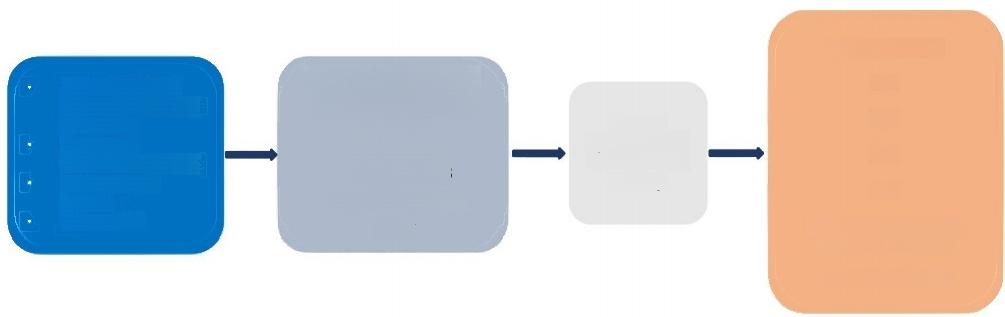


pCR(ypT0/TisypNO) EFS

DFS

**DCR**

**BCR**

Side effects of neoadjuvant therapy (CTCAE.V5.0)

Biomarker indicators

Sintilimab 200mg iv D1 Apatinib 250mg PO

QD1-14 Nab-paclitaxel 125mg/m2,d1,8

CBP AUC 1.5,d1,8

Q3W

II-lllstage(T1c, N1-2orT2-4,NO- 2)TNBC

previously

untreated patients 18-70years、

female

ECOG 0~1

Up to 6

cycles or PD

or intolerable

toxicity

**Figure S6.NeoSAC study flow diagram**

Long-term outcomes will be monitored after initiation of study therapy. Clinical response will be evaluated every 2 cycles before surgery using modified RECIST version 1.1 criteria.After surgery,local pathologists will


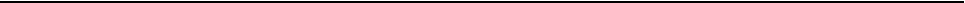
evaluate pathological complete response using the Miller-Payne scoring

system(MP) and residual disease in nodes, along with residual cancer burden (RCB). Adverse events will be monitored throughout the study and for up to 30 days after surgery (90 days for serious adverse events) and graded according to the National Cancer Institute Common Terminology Criteria for Adverse Events version 5.0.

**5. Observation index and observation time**

**5.1 Projects to be completed for inclusion**

**5.1.1 Baseline patient data**

Baseline assessments are done within 7 days before treatment， which included：

1. Basic patient information (including patient code/number in registration database and demographic information);

2. Collect detailed medical history, clarify pathological diagnosis of tumor, understand past treatment history and efficacy, and the current medication status of subjects;

3. Laboratory tests: routine blood tests, routine urine tests, serum biochemistry, tumor markers, thyroid function, cardiac function (BNP, etc.) and virology and other tests when necessary;

4. Electrocardiogram and/or echocardiography, breast and superficiallymph node ultrasound, breast X-ray, breast-enhanced MRI, chest and abdominal CT, head MRI, bone scan, PET-CT if deemed necessary;

5. Physical assessment.

**5.2 Indicators are observed during medication**

**5.2.1 Clinical observation index**

During each medication, subjects' vital signs (body temperature, respiration, blood pressure, and heart rate) will be closely monitored and recorded at any time, as well as local or systemic reactions, chills, high fever, rash, headache,

examination will be performed at every treatment period (21 days), with special attention to the description of primary and surrounding lymph nodes and palpable masses.

**5.2.2 Laboratory and imaging indicators**

Laboratory tests: routine blood tests, routine urine tests, serum biochemistry, tumor markers, thyroid function, cardiac function (BNP, etc.) and virology and other tests when necessary;

Imaging tests: electrocardiogram and/or echocardiography, breast and superficiallymph node ultrasound, breast X-ray, breast-enhanced MRI, chest and abdominal CT, head MRI, bone scan, PET-CT if deemed necessary.

**5.2.3 Biomarkers indicators**

Obtain pre- and post-operative specimens (Preoperative tumor tissue was collected via core biopsies. Postoperative samples were obtained under the guidance of a breast pathologist, with tissue from the tumor bed collected for patients achieving pCR and residual tumor tissue collected for patients with non-pCR) to assess immune therapy response markers (e.g., PD-L1 expression, gene expression signatures) as indicators of pathological response to treatment, as well as changes in the immune microenvironment before and after neoadjuvant therapy.

**6. Evaluation of therapeutic efficiency**

**6.1 Efficacy evaluation criteria**

RECIST1.1(**Table S7**) will be used to evaluate the efficacy of solid tumors.

MP scoring system(**Table S8**) and RCB scoring system(**Table S9**) will be used to evaluate pathological response.

| **Table S7 Evaluation of target lesions RECIST （version 1.1）** | |
| --- | --- |
| Complete Response(CR) | Disappearance of all target lesions.Any pathological lymph nodes (whether target or non-target) must have reduction in short axis to < 10 mm. |

| Partial Response (PR) | At least a 30% decrease in the sum of diameters of target lesions, taking as reference the baseline sum diameters. |
| --- | --- |
| Progressive Disease (PD) | At least a 20% increase in the sum of diameters of  target lesions, taking as reference the smallest sum on study (this includes the baseline sum if that is the  smallest on study). In addition to the relative increase of20%, the sum must also demonstrate an absolute increase of at least 5 mm. (Note:the appearance of  one or more new lesions is also considered progression) |
| Stable Disease (SD) | Neither sufficient shrinkage to qualify for PR nor sufficient increase to qualify for PD, taking as reference the smallest sum diameters while on study. |

| **Table S8 Evaluate pathological response (MP scoring system)** | |
| --- | --- |
| G1 | Invasive cancer cells do not change or only individual cancer cells change, and the number of cancer cells does not decrease in general. |
| G2 | Invasive cancer cells decreased slightly, but the total number is still high, and the reduction of cancer cells do not exceed 30%. |
| G3 | 30% - 90% reduction in invasive cancer cells. |
| G4 | Invasive cancer cells are significantly reduced by more than 90%, and only scattered small clusters of cancer cells or single cancer cells remained. |

| G5 | There are no infiltrating cancer cells in the original tumor bed, but ductal carcinoma in situ may exist. |
| --- | --- |

| **Table S9 Evaluate pathological response (RCB scoring system)** | |
| --- | --- |
| RCB grading | Criteria for judgment |
| RCB 0 | pCR, indicating complete remission of infiltrative lesions. |
| RCB 1 | Partial remission of infiltrative lesions with residual minimal disease evident. |
| RCB 2 | Moderate residual lesions observed, with partial remission of infiltrative lesions. |
| RCB 3 | Extensive residual lesions observed. |

**6.2 Efficacy evaluation index**

**6.2.1 The primary endpoint：**

**pCR rate**: using the definition ofypT0/Tis ypN0 ( namely:no invasive residual in breast or nodes; noninvasive breast residuals allowed) at the time of definitive surgery according to American Joint Committee on Cancer (AJCC) Staging System.

[Time Frame: 4-6 months]

**6.2.2 The secondary endpoints：**

**① Event Free Survival (EFS)**

EFS defined as the duration from randomization to the occurrence of disease progression preventing definitive surgery, local or distant recurrence, the development of a second primary cancer, or death from any cause, whichever come first, is assessed by an investigator blinded to the trial-group assignments. [Time Frame: 2 years]

**② Disease Free Survival (DFS)**

DFS defined as the time from drug onset until recurrence or death for various

reasons.

[Time Frame: 2 years]

**③ Disease Control Rate (DCR)**

CR+PR+SD.

[Time Frame: 4 months]

**④ Rate of BCR**

Rate ofpatients with breast conserving surgery. [Time Frame: 4 months]

**⑤ Rate of BpCR and RCB**

The pCR rate of breast and the overall RCB rate. [Time Frame: 4-6 months]

**⑥ Side effects of neoadjuvant therapy (CTCAE.V5.0)**

[Time Frame: 4 months]

**6.2.3 Exploratory endpoints**

The exploratory outcomes focus on examining the associations between pre- neoadjuvant therapy biomarkers and pCR, as well as the dynamic changes of biomarkers before and after treatment in both the pCR and non-pCR groups. This analysis aimed to elucidate the immunomodulatory effects of neoadjuvant therapy.

**7. Safety capability evaluation**

Safety evaluation includes (CTCAE.V5.0)

**7.1 Adverse Events**

**7.2 Significant Adverse Event**

**7.3 Serious Adverse Events**

**7.4 Adverse Effects**


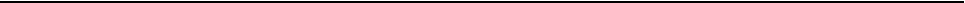
Specific definitions are as follows:

**7.1 Adverse Event (AE)**: unexpected medical conditions or worsening of pre-existing ones may occur during or after drug administration, irrespective of their connection to the studied drug. These unforeseen conditions could manifest as symptoms, signs, or abnormal test results such as laboratory findings or electrocardiograms. In clinical trials, an adverse event encompasses any unanticipated medical condition arising at any point, including during screening or washout phases, even in the absence of treatment investigation.

**7.2 Significant Adverse Event**: any adverse event, excluding serious adverse events, that necessitated the implementation of targeted medical interventions (such as drug withdrawal, dose reduction, and symptomatic treatment), as well as significant abnormalities in hematology or other laboratory tests.

**7.3 Severe Adverse Event (SAE)**: refers to an adverse event that occurs at any phase of the study (including screening or washout, treatment, and follow-up) at any dosage of the study drug or control drug and satisfies one or more of the following criteria:

① Cause death;

② Life-threatening cases caused by experimental drugs;

③ Resulting in hospitalization or prolonged hospitalization;

④ permanent or significant loss of function/disability;

⑤ Teratogenic or carcinogenic.

Some events that require hospitalization or prolonged hospitalization may not be considered serious adverse events, including:

① Hospitalization for social reasons other than adverse events;

② Admission to hospital for elective surgery, examination or other treatment that had been scheduled before entering the trial.

**7.4 Adverse Drug reactions (ADRs)**: discomfort and unexpected reactions are linked to the use of the drug at any dosage. There must be at least a


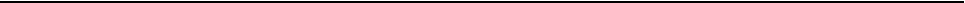
reasonable possibility of a causal relationship between adverse events and

medication. Regarding the severity of adverse reactions, researchers can make assessments based on safety evaluation criteria.

**8. Subsequent therapy**

Postoperative treatment, including radiotherapy and/or chemotherapy, will be determined by the investigator based on clinical guidelines.

**9. Procedures for follow-up and patient data collection**

**9.1 Follow-up set**

The study is expected to last for 2 years.

**9.2 Patient data collection procedures**

Patient information and related data since January 2021. The center will fill out a patient record form indicating all eligible patients and their registration status, and will not be able to collect identifiers such as their real names and ID cards on the patient record form.

All data from the original data file will be summarized in the case Report form or transcribed in the case Report form. All private identifying data will be anonymized to ensure that patients' personal information is not disclosed.

Data collection: Follow-up data will be obtained by research assistants or research nurses during outpatient or inpatient visits, email correspondence, telephone calls, or other web-based communication methods. Patients themselves will provide follow-up data, or in cases where this is not feasible, a relative of the patient will do so.

**10. Data collection**

**10.1 Patient baseline data**

1. Basic patient information (including patient code/number in registration database and demographic information);


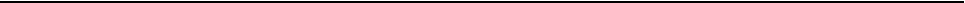
2. Collect detailed medical history, clarify pathological diagnosis of tumor, understand past treatment history and efficacy, and the current medication status of subjects;

3. Laboratory tests: routine blood tests, routine urine tests, serum biochemistry, tumor markers, thyroid function, cardiac function (BNP, etc.) and virology and other tests when necessary;

4. Electrocardiogram and/or echocardiography, breast and superficiallymph node ultrasound, breast X-ray, breast-enhanced MRI, chest and abdominal CT, head MRI, bone scan, PET-CT if deemed necessary;

5. Physical assessment.

**10.2 Data on in-patient or out-patient treatment**

1. Data collection of the first visit

2. Inpatient/outpatient treatment data collection

3. Detailed medication regimen ofpatients

**10.3 Follow-up Data**

1. Treatment information during return visit

2. Clinical efficacy evaluation

3. Clinical safety assessment

4. Laboratory examination

5. Whether to adjust the treatment plan and the adjusted treatment plan

**11. Loss of follow-up and management of cases**

**11.1 Lost to follow-up cases**

A patient who misses one or more routine clinical follow-up visits and cannot be contacted by phone, email, or other available means is considered lost to follow-up. The investigator and study coordinator should make every effort to contact the subjects.

**11.2 Treatment of Lost Cases**

The time and reason of the patient's loss should be recorded in detail and kept in the case report form. Every phone call, email, outpatient follow-up and other forms of contact should be recorded in the case report form. Maintain the integrity of information and store it properly.

**12. Statistic analysis**

**12.1 Statistical analysis plan and statistical software**

Statistical analysis protocols are collaboratively formulated by the head of statistical analysis and the principal investigator. Once all data entry and reviews are finalized, statisticians will conduct comprehensive statistical analyses and generate written reports.

Study Population Definitions: Efficacy will be analyzed in both the intention-to-treat and per-protocol populations. The intention-to-treat group will comprise all enrolled patients who will have received at least one cycle of neoadjuvant therapy, while the per-protocol population will include patients who will have received at least one cycle of neoadjuvant therapy, completed surgery, and undergone pathological evaluation without major protocol violations. Safety analysis will be conducted on the safety population, including enrolled patients who will have received at least one dose of study medication.

The Statistical Analysis Plan (SAP) will provide a detailed description of the analytical methods employed.

**12.2 Data description**

The statistical analysis of all data will be conducted using SPSS software (IBM, Armonk, NY, USA; Version 28.0) and R. Statistical description of all efficacy indicators obtained at each observation time point will be provided. Generally, continuous variables will be statistically described using case number, mean value, standard deviation, quartiles, median, minimum value, and maximum value, while categorical variables will be statistically


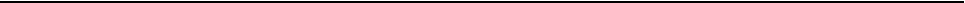
described using the frequency and percentage of each category. The Kaplan-

Meier method will be employed to estimate EFS and DFS. Furthermore, the Kaplan-Meier method and log-rank test will be utilized for univariate assessment of EFS and DFS based on demographic data, baseline clinical information, and toxicity analysis. Additionally, the Cox regression model will be applied to investigate the combined effects of these variables on EFS and DFS in multivariate analysis. Fisher's exact test will be used to assess therapeutic effectiveness, with statistical significance defined as *P*< 0.05.

**13. Statistical Analysis Plan (SAP)**

**13.1 Statistical analysis of primary endpoints**

In this study, the pCR rate after TNBC neoadjuvant therapy will be considered the primary endpoint, defined as the absence of invasive residual disease in the breast or lymph nodes, with no invasive residual breast tissue permitted. (ypT0/TisypN0).

**Analytical method**

The proportion of postoperative pCR in patients with TNBC after 6 cycles of sindilizumab and apatinib combined chemotherapy will be calculated. Kaplan-Meier method will be used to estimate EFS and DFS.

**13.2 Statistical analysis of secondary endpoints**

**13.2.1 Efficacy**

**① pCR**: using the definition ofypT0/Tis ypN0 (namely:no invasive residual in breast or nodes; noninvasive breast residuals allowed) at the time definitive surgery according to AJCC Staging System.

**② EFS**： defined as the time from randomization to occurrence of any event, including disease progression, discontinuation of treatment for any reason or death.

**③ DFS**: refers to the time from the beginning of randomization to disease


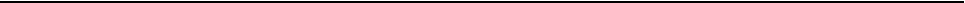
recurrence or death due to disease progression.

**④ DCR**: CR+PR+SD.

**⑤ BCR**: the proportion of malignant tumor surgically removed and breast preserved after TNBC neoadjuvant therapy.

**⑥ Rate of BpCR and RCB**: The pCR rate of breast and the overall RCB rate.

**13.2.2 Safety**

Adverse events: adverse events are coded according to the international dictionary of medical terms (MedDRAV20.0).

Adverse events: adverse events that are "related", "likely related", or "possibly related" to the drug under study.

Summary of adverse events by group, including all adverse events, serious adverse events, adverse reactions, serious adverse reactions, adverse events leading to death, and adverse events leading to withdrawal of study drugs; Adverse events and adverse reactions will be summarized according to severity and correlation with the drugs under study. Adverse events and adverse reactions will be summarized by organ system category (SOC) and preferred term (PT). The list lists all adverse events.

**14. Interim analysis**

In order to ensure the efficacy and safety of the study and maximize the protection of subjects' rights and interests, the study plans to enroll patients in two stages.

We use a Simon ’ s minimax two-stage design with a one-sided α error of 5% and a power of 80% to estimate the pCR rate as the primary endpoint. We hypothesize that the addition of apatinib to sintilimab combined with carboplatin plus nab-paclitaxel chemotherapy improves the pCR rate from 43.5% (based on the NeoTRIPaPDL1 results reported in 2019) to 66.0%. The target accrual is a minimum of 16 patients in the first stage, and if responses are confirmed in more than 7 patients, 14 additional patients will be accrued


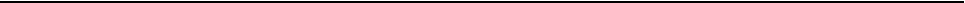
in the second stage, with a total of 30 patients. Assuming a 10% drop-out rate,

we estimate a total sample size of 34 patients.

**15. Statistical analysis report**

Will be done by professional statisticians. Form a complete written report after the statistics.

**16. Research indicators of ethics and administration**

Ethics Committee (EC) or Institutional Review Board (IRB)

This study shall comply with the Good Clinical Practice (GCP) principles of the Declaration of Helsinki, ICH-GCP and CFDA. The investigator must be approved by an independent ethics committee. The collection of patient data requires confidential treatment of patient privacy data. If the disclosure does not involve patient privacy, the research sponsor shall apply for approval documents from the ethics committee/institutional Review Board in the corresponding institution.

This study has been certified by the Ethics Committee of the Affiliated Hospital of Qinghai University (P-SL-2020078).

**17. Human Genetic resources record**

This study has been registered on the Chinese Human Genetic Resources Management Platform

**18. Confidentiality**

All records relating to patient identity are confidential and will not be made public to the extent permitted by relevant laws and/or regulations. Only patient numbers and initials are recorded in the form. If the patient's name appears in any other document (e.g., pathology report), it must be withheld before a copy of the document is provided to the sponsor. Research reports


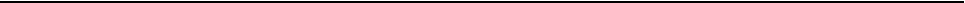
stored by computer must comply with local data protection laws.

Researchers will keep a record that will accurately identify each patient enrolled in the registry.

**Privacy and Confidentiality Principle**

No individual or organization will be allowed to access your medical records, except for doctors, nurses, medical administrations, ethics committees, and those permitted by law. Your personal information will not be disclosed in any public report on the results of this follow-up. We will strive to protect the privacy of your personal medical information to the extent permitted by law.

**19. Information and informed consent of selected patients**

Core study information and informed consent will be provided. Prior to the initiation of the study, the investigator must obtain approval or clearance from the Ethics Committee/Institutional Review Committee for the written informed consent and other materials to be provided to the patient. The written approval from the IRB/Institutional Review Board and the approved information for enrolled patients/informed consent must be archived in the study documentation. Written informed consent, including consent for optional biomarkers, must be obtained before any specific study procedures are performed. Dates of study participation and signed informed consent from enrolled patients should be accurately recorded in their medical records. Before enrolling each patient in the study, it is the responsibility of the study physician to provide them or their designated representative with a comprehensive written description of the study's purpose, procedures, potential risks, and benefits, and to obtain their authorization to use and/or disclose personal and/or health data. The patient's informed consent shall be in duplicate, with one copy provided to the patient for their own records. If multiple centers participate in the study, the consistency of data collected


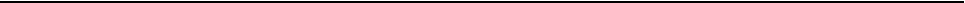
from the medical record form at each center will be verified, and any

discrepancies will be addressed by the physician for clarification. Participation in follow-up or withdrawal is voluntary. The decision to participate in the study is entirely up to the individual, and they may refuse to participate or withdraw at any time during the follow-up without any negative impact on their relationship with the doctor or on any medical or other benefits they may receive.

**20. Data administration**

1. Any information entered in the medical record form must be consistent with the original medical record.

2. Study documents and all original materials shall be kept in accordance with local laws and regulations or guidelines.

3. The researcher will fill the collected data into the medical record report form patient management system according to the research protocol.

**21. Attend follow-up visits for possible risks**

**21.1 Risk of disclosure of clinical data and personal information**

This risk can be reduced or avoided by the following measures: the patient record form will not collect the real name of the patient, identification card and other identifiers. All privately identified data will be anonymized. The case report form will be managed in strict confidence and cannot be read by non-medical staff or the patient himself. The pathology report form should not be borrowed, and strict procedures should be handled when reading it. Publishing research data to hide personal information.

**21.2 Contact is intrusive and inconvenient**

They can ask for the most convenient way to contact them, such as mobile phone, SMS, email, landline, fax, or letter, and contact at the most convenient time they request.


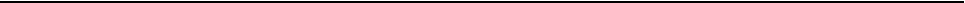


**22. Possible benefits of participating in clinical trials**

Participating in this study holds the promise of yielding improved outcomes from tailored treatment regimens (Table S10). The insights gleaned are poised to offer invaluable support not only to the participants themselves but also to others grappling with similar afflictions. Should the findings align with expectations, both the participants and fellow sufferers stand to reap significant benefits.

**Table S10 This study is a partially free drug and testing program**

| Drug | Free Medication | Free testing items |
| --- | --- | --- |
| Sintilimab | Non-poor patients: buy three cycles, get three free cycles; Individuals with low income:  6 cycles free. | WES, RNA-seq, PD-  L1, CD8, TMB, mIF,  etc. |
| Apatinib | All patients: buy two boxes of  pills, send two boxes of pills,  until 6 cycles. |  |

**23. Related expenses and compensation**

**23.1 Related expenses**

In the entire treatment process, participants will require regular phlebotomy for routine blood tests, blood biochemistry assessments, nuclear medicine tumor marker evaluations, nuclear medicine procedures, cardiac function assessments, and laboratory examinations, along with imaging evaluations such as ultrasound, CT scans, MRI scans, etc. These evaluations will continue until the completion of the study, either upon disease progression or if the participant experiences intolerable toxicity leading to the withdrawal of treatment either voluntarily or upon the recommendation of their physician. All visits will be scheduled as part of the routine protocol, and no additional follow-up visits or examinations are anticipated, thus eliminating any extra travel expenses for participants.


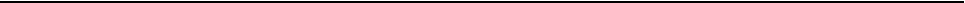
The follow-up services to be included in this study will be provided free of

charge. These services will encompass telephone consultations, establishment of case report forms, transmission of result information, and more. Study follow-up doctors will reach out to participants via phone, and participants will not be required to pay any fees, including phone charges.

**23.2 Compensation**

Commercial insurance (China Pacific Insurance (Group) Co., LTD.) will be procured for all patients participating in the study. In the event that participants experience additional risks or adverse reactions attributable to the study, the insurance company will assume full responsibility for all compensation. This arrangement absolves the researchers and the study institution of any liability in such instances.

**24. References**

[ 1] Siegel RL,Miller KD,Jemal A,Cancer statistics, 2019.[J] .CA Cancer J Clin, 2019, 69: 7-34.

[2] Onitilo AA,Engel JM,Greenlee RT, et al. Breast cancer subtypes based on ER/PR and Her2 expression: comparison of clinicopathologic features and survival.[J] .Clin Med Res, 2009, 7: 4-13.

[3] Brenton JD,Carey LA,Ahmed AA, et al. Molecular classification and molecular forecasting of breast cancer: ready for clinical application?[J] .J Clin Oncol, 2005, 23: 7350-60.

[4] Mayer IA,Abramson VG,Lehmann BD, et al. New strategies for triple-negative breast cancer-- deciphering the heterogeneity.[J] .Clin Cancer Res, 2014, 20: 782-90.

[5] Pareja F,Reis-Filho JS,Triple-negative breast cancers - a panoply of cancer types.[J] .Nat Rev Clin Oncol, 2018, 15: 347-348.

[6] Wolff AC,Hammond MEH,Allison KH, et al. Human Epidermal Growth Factor Receptor 2 Testing in Breast Cancer: American Society of Clinical Oncology/College of American Pathologists Clinical Practice Guideline Focused Update.[J] .J Clin Oncol, 2018, 36: 2105-2122.

[7] den Brok WD,Speers CH,Gondara L, et al. Survival with metastatic breast cancer based on initial presentation, de novo versus relapsed.Breast Cancer Res Treat 2017, 161(3):549-556.

[8] Gobbini E, Ezzalfani M, Dieras V, et al. Time trends of overall survival among metastatic breast cancer patients in the real-life ESME cohort. Eur J Cancer. 2018 Jun;96:17-24.

[9] Lehmann BD,Bauer JA,Chen X, et al. Identification of human triple-negative breast cancer subtypes and preclinical models for selection of targeted therapies.[J] .J Clin Invest, 2011, 121: 2750- 67.

[ 10] Jiang YZ,Ma D,Suo C, et al. Genomic and Transcriptomic Landscape of Triple-Negative Breast Cancers: Subtypes and Treatment Strategies.[J] .Cancer Cell, 2019, 35: 428-440.e5.

[ 11] Spring LM,Fell G,Arfe A, et al. Pathologic Complete Response after Neoadjuvant Chemotherapy and Impact on Breast Cancer Recurrence and Survival: A Comprehensive Meta-analysis.[J] .Clin Cancer Res, 2020, 26: 2838-2848.

[ 12] von MG,Untch M,Blohmer JU, et al. Definition and impact of pathologic complete response on prognosis after neoadjuvant chemotherapy in various intrinsic breast cancer subtypes.[J] .J Clin Oncol, 2012, 30: 1796-804.

[ 13] Untch M,Jackisch C,Schneeweiss A, et al. Nab-paclitaxel versus solvent-based paclitaxel in neoadjuvant chemotherapy for early breast cancer (GeparSepto-GBG 69): a randomised, phase 3 trial.[J] .Lancet Oncol, 2016, 17: 345-356.

[ 14] Sikov WM,Berry DA,Perou CM, et al. Impact of the addition of carboplatin and/or bevacizumab to neoadjuvant once-per-week paclitaxel followed by dose-dense doxorubicin and cyclophosphamide on pathologic complete response rates in stage II to III triple-negative breast cancer: CALGB 40603 (Alliance).[J] .J Clin Oncol, 2015, 33: 13-21.

[ 15] Hahnen E,Lederer B,Hauke J,et al. Germline Mutation Status, Pathological Complete Response, and Disease-Free Survival in Triple-Negative Breast Cancer: Secondary Analysis of the GeparSixto Randomized Clinical Trial.[J] .JAMA Oncol, 2017, 3: 1378-1385.

[ 16] von MGr,Schneeweiss A,Loibl S, et al. Neoadjuvant carboplatin in patients with triple-negative and HER2-positive early breast cancer (GeparSixto; GBG 66): a randomised phase 2 trial.[J] .Lancet Oncol, 2014, 15: 747-56.

[ 17] Yuan Y,Lee JS,Yost SE, et al. Phase II Trial of Neoadjuvant Carboplatin and Nab-Paclitaxel in Patients with Triple-Negative Breast Cancer.[J] .Oncologist, 2020, undefined: undefined.

[ 18] Roodhart JM,Langenberg MH,Witteveen E, et al. The molecular basis of class side effects due to treatment with inhibitors of the VEGF/VEGFR pathway.[J] .Curr Clin Pharmacol, 2008, 3: 132-43.

[ 19] Syrigos KN,Karapanagiotou E,Boura P, et al. Bevacizumab-induced hypertension: pathogenesis and management.[J] .BioDrugs, 2011, 25: 159-69.

[20] Liu J,Liu Q,Li Y, et al. Efficacy and safety of camrelizumab combined with apatinib in advanced triple-negative breast cancer: an open-label phase II trial.[J] .J Immunother Cancer, 2020, 8: undefined.

[21] Schmid P,Rugo HS,Adams S, et al. Atezolizumab plus nab-paclitaxel as first-line treatment for unresectable, locally advanced or metastatic triple-negative breast cancer (IMpassion130): updated efficacy results from a randomised, double-blind, placebo-controlled, phase 3 trial.[J] .Lancet Oncol, 2020, 21: 44-59.

[22] Mittendorf EA,Zhang H,Barrios CH, et al. Neoadjuvant atezolizumab in combination with sequential nab-paclitaxel and anthracycline-based chemotherapy versus placebo and chemotherapy in patients with early-stage triple-negative breast cancer (IMpassion031): a randomised, double-blind, phase 3 trial.[J] .Lancet, 2020, 396: 1090-1100.

[23] Schmid P,Cortes J,Pusztai L, et al. Pembrolizumab for Early Triple-Negative Breast Cancer.[J] .N Engl J Med, 2020, 382: 810-821.

**25. Protocol Amendment**


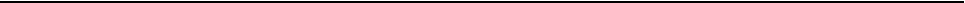
1. The dose of carboplatin was reduced from an AUC of 2.0 to an AUC of

1.5 due to a high frequency of over grade 3 neutropenia, which was approved by institutional review boards on May 13, 2021.

2. Due to the impact of the COVID-19 pandemic, the enrollment rate for this study was slower than expected, prompting a modification to the protocol. Therefore, in January 2023, we revised the anticipated enddate to February 29, 2024, and obtained approval from the Ethics Committee.
